# The Key to the Extraordinary Thermal Stability of *P. furiosus* Holo-Rubredoxin: Iron Binding-Guided Packing of a Core Aromatic Cluster Responsible for High Kinetic Stability of the Native Structure

**DOI:** 10.1371/journal.pone.0089703

**Published:** 2014-03-06

**Authors:** Satya Prakash, Monica Sundd, Purnananda Guptasarma

**Affiliations:** 1 Department of Biological Sciences, Indian Institute of Science Education and Research (IISER), Mohali, SAS Nagar, Punjab, India; 2 Protein Engineering Division, Institute of Microbial Technology (IMTECH), Chandigarh, India; 3 NMR Laboratory, National Institute of Immunology (NII), New Delhi, India; Aligarh Muslim University, India

## Abstract

*Pyrococcus furiosus* rubredoxin (PfRd), a small, monomeric, 53 residues-long, iron-containing, electron-transfer protein of known structure is sometimes referred to as being the most structurally-stable protein known to man. Here, using a combination of mutational and spectroscopic (CD, fluorescence, and NMR) studies of differently made holo- and apo-forms of PfRd, we demonstrate that it is not the presence of iron, or even the folding of the PfRd chain into a compact well-folded structure that causes holo-PfRd to display its extraordinary thermal stability, but rather the correct iron binding-guided packing of certain residues (specifically, Trp3, Phe29, Trp36, and also Tyr10) within a tight aromatic cluster of six residues in PfRd's hydrophobic core. Binding of the iron atom appears to play a remarkable role in determining subtle details of residue packing, forcing the chain to form a hyper-thermally stable native structure which is kinetically stable enough to survive (subsequent) removal of iron. On the other hand, failure to bind iron causes the same chain to adopt an equally well-folded native-like structure which, however, has a differently-packed aromatic cluster in its core, causing it to be only as stable as any other ordinary mesophile-derived rubredoxin. Our studies demonstrate, perhaps for the very first time ever that hyperthermal stability in proteins can owe to subtle differences in residue packing *vis a vis* mesostable proteins, without there being any underlying differences in either amino acid sequence, or bound ligand status.

## Introduction

Hyperthermostable proteins from hyperthermophile organisms generally unfold irreversibly, owing to the existence of high energy kinetic barriers that separate the protein's native state from its unfolded states and prevent both facile unfolding, and refolding [1]–[4]. However, certain exceptions have been reported [5]–[7]. One relates to *Pyrococcus furiosus* rubredoxin (PfRd), an iron-sulphur cluster-containing protein reported to refold both in the presence [7], and absence of iron [8], [9]. PfRd contains a single inorganic iron atom bound in tetrahedral geometry to the sulfur atoms of four cysteine residues in its sequence [numbered Cys5, Cys8, Cys38 and Cys41] through non-covalent coordinate bonds [10], [11]. It is a small, monomeric protein containing only 53 residues. However, PfRd displays such a high degree of structural (thermal) stability that it has been referred to as being perhaps ‘the most structurally-stable protein known to man’ [3].

PfRd's iron-containing (holo) and iron-lacking (apo) forms have been reported to display virtually identical secondary structural contents, and their structures are thought to be identical. However, the two forms have also been reported to display profoundly different structural stabilities [8], [9]. Holo-PfRd cannot be unfolded by simple exposure to either extreme temperature alone, or to extreme concentrations of denaturants alone, at room temperature. Our own unpublished observations show that holo-PfRd undergoes partial unfolding, extremely slowly (over a time period of several days) only if it is subjected simultaneously to a combination of extreme temperature (i.e., >90°C and up to 105°C) and extreme denaturant concentration (∼6 M guanidium hydrochloride). Alternatively, as the literature suggests, the protein can also be destabilized by exposure to high concentrations of trichloriacetic acid in the presence of denaturant over long durations of exposure [8], [9]. In sharp contrast to what is seen with holo-PfRd, iron-lacking PfRd (i.e., Apo-PfRd) made by refolding acid-unfolded, iron-depleted PfRd in the absence of iron, turns out to display only a very ordinary degree of thermal stability like that of any moderately stable mesophile protein. Apo-PfRd undergoes immediate and facile unfolding upon exposure to any temperature above 70°C in the absence of denaturant, or upon exposure to 6 M guanidium hydrochloride (Gdm.HCl), at room temperature [8]. The existence of such profound differences between the structural stabilities of holo-PfRd and Apo-PfRd have been held to be highly intriguing from a structural-biochemical viewpoint, because the two forms of PfRd have virtually identical secondary structural contents and seemingly overlapping near- and far-UV circular dichroic (CD) spectra. However, in the absence of high-resolution information about any subtle structural differences between the two forms, it has seemed as if holo-PfRd's extreme stability must owe wholly to the presence of the iron atom (and the consequently formed iron-sulfur cluster) in its structure, since this is the only obvious feature distinguishing it from Apo-PfRd. This simple view has been strengthened by the report that a cysteine-lacking mutant of PfRd, in which all cysteine residues have been replaced by serine, is indistinguishable from Apo-PfRd in terms of its CD spectra and structural stability [9].

PfRd's iron-sulfur cluster thus clearly plays a major role in causing it to be extraordinarily stable. However, there is a problem with the assumption that the secret lies entirely in the presence of the iron atom, and the thermodynamic stabilization offered to the protein by the iron-sulfur cluster. Such a simplistic view neglects the possibility that Apo-PfRd adopts a structure that is so subtly different from that of holo-PfRd that it is impossible for low-resolution spectroscopic approaches like circular dichroism (CD) or fluorescence spectroscopy to detect any differences. If this were indeed the case, the role of the iron atom could be not to provide stability, but rather to help the PfRd molecule to attain a structure with a particular packing scheme of residues critical to determining its thermodynamic and kinetic structural stability. Lack of iron-binding during folding could result in a structure with a profoundly lower stability owing to subtle differences in residue packing. Clearly, such alternative explanations cannot be summarily ruled out without high-resolution investigation of side chain packing within the structures of Apo-PfRd and holo-PfRd.

This is the lacuna that we have addressed in this paper, using various approaches including NMR spectroscopy and two different apo forms of PfRD. To distinguish between these apo forms, hereinafter, we refer to the Apo-PfRd created by refolding PfRd in the absence of iron as ‘Apo-1 PfRd’. In contrast, we refer to an apo form created by teasing iron out of holo-PfRd without effecting any unfolding as ‘Apo-2 PfRd’. We report that Apo-2 PfRd is extraordinarily hyperthermostable, like holo-PfRd, and that it does not transform into Apo-1 PfRd, suggesting that the native structure formed upon iron binding is kinetically stable and can survive the removal of iron. Detailed characterization using CD, fluorescence and NMR suggests that while all three forms, i.e., holo-PfRd, Apo-2 PfRd and Apo-1 PfRd are identical in most structural respects, an aromatic cluster in the protein's core comprising mainly residues Trp3 (W3), Tyr10 (Y10), Phe29 (F29), and Trp36 (W36), but also two other aromatic residues, happens to be structured differently in Apo-1 PfRd, and in holo-PfRd/Apo-2 PfRd. Interestingly, theoretical (quantum chemical) considerations suggest that PfRd's hyperthermal stability must owe in part to this very same aromatic cluster [12].

Our study thus establishes that the presence of iron in holo-PfRd is not singularly responsible for its extraordinary stability. Our results also reconcile all available information about PfRd's stability together into a cogent thesis, by (i) providing a novel and fascinating insight into iron's role in determining residue packing in PfRd's core, and also (ii) providing experimental support for pre-existing arguments concerning the importance of PfRd's aromatic cluster to the molecule's hyperthermal stability. To the best of our knowledge, this is the first study which shows that subtle differences in residue packing (in otherwise indistinguishable structures) can bring about profound differences in stability, and transform an ordinary protein into a highly kinetically-stable protein.

## Materials and Methods

### Stock solutions

Ferrozine was obtained from Sigma (catalog no. P9762); Fe2+ iron was obtained through dissociation of FeSO4.7H2O obtained from Merck (catalog no. 61751005001730); guanidine hydrochloride (Gdm.HCl) was obtained from Promega (catalog no. H5383); beta-mercaptoethanol was obtained from Sigma (catalog no. M7522). Stock solutions of ferrozine (100 mM) and FeSO4.7H2O (18.7 mM) were prepared in water. A stock solution of PfRd was prepared at a concentration of 30.0 mg/ml in sodium phosphate buffer (pH 7.2; 20 mM). A stock solution of Gdm.HCl (8 M) was prepared in sodium phosphate buffer (pH 7.2; 20 mM).

### PfRd protein

#### Cloning

A gene encoding PfRd was synthesized through splicing by overlap extension PCR (SOE-PCR) using overlapping oligonucleotides to generate a DNA sequence encoding PfRd's amino acid sequence, optimized for expression in *E.coli*. The gene was first cloned into the vector pQE-30 (Qiagen) between the cloning sites, BamH1 and HindIII, and the resultant plasmid was transformed into XL1 blue *E.coli* strain cells, which were grown in LB media in the presence of the antibiotics, tetracyclin (0.0125 mg/ml) and ampicillin (0.1 mg/ml) at 37°C for 14 to 16 hours using shaking at 200 rpm. The plasmid was isolated from these cells and sequenced to confirm that the gene had been synthesized correctly.

#### Expression

The pQE-30 plasmid carrying the gene insert was transformed into the M15 pRep-4 strain of *E.coli* for protein expression. Transformed cells were grown in LB media in the presence of the antibiotics, kanamycin (0.025 mg/ml) and ampicillin (0.1 mg/ml) at 37°C, with shaking at 200 rpm. The primary culture was grown for 10 to 14 hours, following which a 1% inoculum was used to grow a secondary culture which was induced with 1 mM IPTG at a culture OD_600_ value of 0.5 and grown for 5 hours, prior to harvest through centrifugation at 5000 rpm for 10 min at 4°C. Harvested cells were sonicated using alternate ON (15 seconds) and OFF (10 seconds) pulses on ice for 30–50 minutes on a sonicator in lysis buffer (NaH2PO4 50 mM; NaCl 300 mM; Imidazole 10 mM; pH 8.0 using NaOH) until the released PfRd protein (red in color, due to its oxidized bound iron) became visible in the lysate undergoing sonication.

#### Purification

Ni-NTA affinity chromatography (under native conditions) was employed for purification. The cell lysate (∼20 ml) was loaded on to a Ni-NTA column (∼2 ml resin volume), and the column was washed with NaH2PO4 50 mM, NaCl 300 mM, Imidazole 20 mM, pH 8.0 using NaOH. Protein was eluted with NaH2PO4 50 mM, NaCl 300 mM, Imidazole 250 mM, pH 8.0 using NaOH.

#### Removal of imidazole

The eluted protein was dialyzed with a 1.2 kDa cut off membrane on a Biodialyzer (Sigma) against sodium phosphate buffer (20 mM, pH 7.2), to remove imidazole.

#### Concentration

The dialyzed protein was concentrated using a centrifugal concentration device (1–3 kDa cut-off, Amicon). The obtained PfRd protein had the following characteristics: Length of 65 amino acids, including a 12-residue N-terminal affinity tag; Molecular weight of 7277.50 Da; Molar extinction coefficient at 280 nm of 14300, corresponding to an O.D of 1.0∼0.51 mg/ml PfRd. The protein was red in color due to the presence of the iron atom in the oxidized state. It was calculated to have a pI of 5.1.

### Spectroscopy and spectrometry

#### Circular dichroism

Far-UV and near-UV CD spectra were collected on a Jasco J-810 CD spectropolarimeter using cuvettes of 1 cm/0.5 cm/0.2 cm path length with flushing of nitrogen gas at ∼30 litres/min. Raw ellipticity data was collected in the range of 250 to 180/190/200 nm. Data for raw ellipticity (θ_obs_) was converted to mean residue ellipticity, [θ], using the formula, [θ, in millidegrees] = [θ_obs_(in millidegrees)×MRW×100]/[concentration (mg/ml)×path length (cm)], where MRW stands for mean residue weight, which is ∼112 for PfRd. Spectra are shown after standard 5-point averaging-based smoothing. Some far-UV spectra show both negative bands of PfRd (discussed in the Results and Discussion section), while most spectra show only the longer wavelength negative band arising from the beta and alpha secondary structures and aromatic contributions.

#### UV/Visible absorption spectroscopy

Absorption spectroscopy was performed on a Cary 50 or Jasco V-650 UV-Visible spectrophotometer. Absorbance scans were performed from 200–600 nm according to need.

#### Fluorescence spectroscopy

Fluorescence spectroscopy was carried out on a Jasco J810 CD spectropolarimeter fitted with a FMS827 emission monochromator fluorescence accessory. For fluorescence emission spectra of proteins, excitation wavelength was set at 285/290 nm, with a bandpass of 5 nm and the emission spectrum was recorded from 300–400 nm with a bandpass of 5 nm.

#### NMR spectroscopy

NMR samples consisted of 2 mM protein, in 20 mM sodium phosphate buffer, pH 6.0, 100 mM NaCl, 90% H_2_O, 10% D_2_O. Two dimensional NMR spectra, i.e., ^1^H TOCSY, and ^1^H NOESY spectra, were acquired on a BrukerAvance III 700 MHz spectrometer, equipped with a TCI cryoprobe installed at the National Institute of Immunology, New Delhi, India using Bruker pulse sequences. Experiments were performed at 298K throughout. NMR data was processed on a workstation running Red Hat Enterprize Linux 5.0, using NMRPipe/NMRDraw [13] and analyzed using Sparky (T. D. Goddard and D. G. Kneller, SPARKY 3, University of California, San Francisco; unpublished method). The data was multiplied by a phase shifted sinebellapodization function in all dimensions.^1^H TOCSY experiment spectra were collected with 1024 (t2)×512 (t1) data points and a mixing time of 60 msec. In the ^1^H NOESY experiments, a mixing time of 150 msec was used, with 1024 (t2)×512 (t1) data points. Spectra were referenced using Sodium 2, 2-dimethyl-2-silapentane-5-sulfonate (DSS) as a chemical shift standard. *Mass spectrometry.* MALDI-TOF mass spectra were acquired on an AB-SCIEX Voyager DE-STR mass spectrometer, using standard methods.

### The making of Apo-1

The method of making this particular apo form of PfRd (Apo-1) has already been described by other workers [8]. However, below we briefly describe our own adaptation of this method. A solution of native PfRd in sodium phosphate buffer (20 mM, pH 8.0) was added to an equal volume of 50% TCA (w/v), 143 mM beta-mercaptoethanol (B-ME) and 50 mM EDTA in sodium phosphate buffer (20 mM, pH 8.0), such that the final concentration of TCA was 25% (w/v). The solution was mixed vigorously to generate a white precipitate. Centrifugation was done at 14500 rpm for 20 min to pellet the white precipitate (PfRd protein). This process was repeated through cycles of re-suspension of the pellet in a solution of 50% (w/v) TCA, 143 mM beta-mercaptoethanol and 100 mM EDTA in sodium phosphate buffer (20 mM, pH 8.0), vigorous mixing, and centrifuging at 14500 rpm for 20 min each time. After 3–4 such cycles, the white pellet was dissolved in a solution of 5 M Gdm.HCl, 143 mM beta-mercaptoethanol and 100 mM EDTA in sodium phosphate buffer (20 mM, pH 7.2). This solution, containing completely unfolded protein, was dialyzed extensively against a solution of 143 mM beta-mercaptoethanol, 220 mMNaCl in sodium phosphate buffer (55 mM, pH 6.5) to allow chain refolding to occur. Buffer was exchanged by dialyzing extensively against sodium phosphate buffer (20 mM, pH 7.2) having 3 mM EDTA. Final concentration of EDTA was maintained at 3 mM, in order to chelate away any trace amount of iron. The resultant protein was the apo form of PfRd which we refer to as Apo-1.

### The making of Apo-2

Our method of making Apo-2 consists of several smaller steps and procedures, each of which is explained below. Briefly, Apo-2 was generated by placing the protein in a concentration of Gdm.HCl (6 M) which elicits no detectable conformational effects at room temperature, but which could potentially ‘loosen-up’ the iron-binding region sufficiently to allow access of small redox reagents to the iron atom in PfRd which normally exists in an oxidized state. In the presence of 6 M Gdm.HCl, beta-mercaptoethanol was then used as a reducing agent to access and reduce the iron atom in PfRd. The reduced iron was then chelated out of PfRd through use of ferrozine which is well-known to chelate reduced. Finally, gel filtration chromatography was used to separate out the ferrozine-bound iron from free ferrozine and from the apo form of PfRd thus generated (Apo-2), in order to demonstrate that iron was indeed released by PfRd and taken up by ferrozine. The absorption band due to iron in PfRd was monitored to confirm iron-loss. Separately, the protein was treated in a manner that would release and reduce the iron, with ferrozine present to detect reduced iron colorimetrically, to establish whether any trace iron remained. Details are given below, in a systematic manner.

#### (i) Confirming that mercaptoethanol reduces iron

Beta-mercaptoethanol (B-ME, or 2-mercaptoethanol) is a reducing agent commonly used to reduce disulphide bonds in proteins. It is also reported to reduce ferric iron (Fe3+) in mines, both alone and in presence of certain additives [Beard, R. N., Vinson, E.F., U.S. patent 6060435]. The reduction of ferric to ferrous iron (Fe^2+^) proceeds via a complex formation of the ferric iron with beta-mercaptoethanol with or without inorganic sulphides (using inorganic sulphides may help complete the six coordinate bonds of iron). We considered it appropriate to describe some experiments here further establishing the reduction of inorganic iron by beta-mercaptoethanol. Control experiments are shown in Figure S1, Panel A, in File S1. Addition of ferrozine to freshly prepared aqueous ferrous sulphate, FeSO4.7H2O, leads to immediate formation of a magenta color (tube 4; Figure S1, panel B, in File S1). This is not seen upon addition of ferrozine to aqueous ferric chloride, FeCl3 (tube 1; Figure S1, panel B, in File S1). When beta-mercaptoethanol is added to FeCl3, ferrozine addition leads to the appearance of magenta color (tube 2; Figure S1, panel B, in File S1). There is no effect of the presence of 6 M Gdm.HCl upon the reducing ability of beta-mercaptoethanol (tube 3; Figure S1, panel B, in File S1), establishing that this denaturant can be used to denature iron-containing proteins without affecting either the reducing ability of beta-mercaptoethanol or the generation of color by ferrozine binding to ferrous ion.

#### (ii) Using beta-mercaptoethanol to reduce PfRd's iron atom and ferrozine to chelate the reduced iron

A solution of native PfRd consisting of 6 M Gdm.HCl, 14.3 mM beta-mercaptoethanol and 10 mM ferrozine from Sigma (catalog no. P9762) in sodium phosphate buffer (20 mM, pH 7.2) was prepared. The final concentration of PfRd was 1.0 mg/ml. After a few minutes, magenta colour appeared, indicating that ferrozine had bound to the released (reduced) iron. This solution was kept at room temperature for 48 hours. The magenta coloured complex was removed from the protein solution by gel filtration chromatography, as described below.

#### (iii) Using chromatography to demonstrate iron transfer from PfRd to ferrozine, to generate Apo-2 PfRd

A Superdex Peptide 10/300GL gel filtration chromatographic column from GE Healthcare (catalog no. 17-5176-01) was used on an Akta Purifier-10 automated chromatographic workstation. The Superdex Peptide resin performs optimum separation in the range of 100–7,000 Da. The column was equilibrated with sodium phosphate buffer (pH 7.2; 20 mM). To generate the sample for chromatography, a solution was first created by mixing PfRd (34 µl; 30 mg/ml), ferrozine (100 µl, 100 mM), beta-mercaptoethanol (1 µl, 14.3 M), and Gdm.HCl (750 µl, 8M). The volume was made up to 1000 µl using water. Of this, 500 µl was loaded onto the column, with monitoring done at 280 nm (aromatic absorption band) and 490 nm (iron-sulphur cluster's absorption band) to observe elution of the protein, and monitoring at 560 nm to observe elution of reduced iron-bound ferrozine. The beta-mercaptoethanol was added to reduce all released iron, as it is well known that this reagent reduces iron. The key data in this regard is presented in Figures S2a–S2d in File S1 which explain our novel method for chromatographic separation of Apo2-PfRd (Apo-2) from the ferrozine-bound iron released by the native holoPfRd.

### Aromatic replacement mutations in PfRd

#### Materials used

QuickChange Site-Directed Mutagenesis Kit, containing PfuTurbo™ DNA polymerase (2.5 U/µl), 10× reaction buffer, Dpn I restriction enzyme (10 U/µl), pWhitescript™ 4.5-kb control plasmid (5 ng/µl), dNTP mix, *Epicurian coli* XL1-Blue supercompetent cells,pUC18 control plasmid (0.1 ng/µl in TE buffer) and control primers, was purchased from Stratagene (product code #200518).

All primers were designed by the guidelines given in the kit's manual and obtained from Integrated DNA Technologies (IDT) Inc., Coralville, IN, USA. The primers were dissolved in suitable amount of TE buffer pH 8.0 to give final stock concentration of 125 µM. The list of the forward (F) and reverse (R) primers used for PCR mutagenesis is given below. The final concentration of primers used in QuickChange Site-Directed Mutagene sis Kit's protocol was 125 ng/µl. The primer concentration for Splicing by overlap extension PCR was 125 µM. The primer sequences are mentioned below : Primers for W3A, 1F-5′-CACGGATCCGCTAAAGCTGTTTGC-3′, 1R-5′-CCACAGATTTTGCAAACAGCTTTAGCGG-3′; Primers for Y10A, 4F-5′-GGTTTGCAAAATCTGTGGAGCTATCTACG-3′, 4R-5′-CCAGCGTCTTCGTCGTAGATAGCTCCAC-3′; Primers for Y12A, 6F-5′-GCAAAATCTGTGGATACATCGCTGACGAAG-3′, 6R-5′-GTCACCAGCGTCTTCGTCAGCGATGTATC-3′; Primers for F29A, 9F-5′-CCCCGGGTACCAAAGCTGAAGAAC-3′, 9R-5′-GTCAGGCAGTTCTTCAGCTTTGGTAC-3′; Primers for W36A, 10F-5′-GAAGAACTGCCTGACGACGCTGTTTGTC-3′, 10R-5′-CCACAGATCGGACAAACAGCGTCGTCAG-3′; Primers for F48A, 15F-5′-CCAAAATCCGAAGCTGAAAAACTG -3′, 15R-5′-GTCTTCCAGTTTTTCAGCTTCGGA-3′

Mutations done by PCR using QuickChange Site-Directed Mutagenesis Kit: Composition : Buffer = 5 µl; Template = 1 µl(26 ng); Forward primer = 1 µl; Reverse primer = 1 µl; dNTP mix = 1 µl; PfuTurbo enzyme = 1 µl; Deionized water = 41 µl. (Total volume 51 µl). Cycles: PfuTurbo provided with the kit was claimed to replicate DNA at the speed of 1 min per kb. The size of the template (pQE30+gene) was 3.566 kb. Therefore, the extension time was given ∼4 min. Cycling was done as 95°C = 30 seconds; 95°C = 30 seconds; 55°C = 1 min; 68°C = 4 min;(total no. of cycles = 20); 68°C = 10 min; 4°C = hold. Dpn digestion: Added 0.6 µl to 26 µl to the reaction sample and incubated at 37°C for 1 hr. 5 µl of the final product was transformed in chemical competent *E. coli* XL1 blue cells by heat shock method. Mutants W3A, Y10A and Y12A were made by the same protocol.

The other three mutants were made by splicing by overlap extension (SOE) PCR. The final product obtained was digested by BamH1 and HindIII enzymes and Quick Ligated in pQE30, digested by same enzymes. The product was gel purified and transformed in XL1Blue E.coli Cells. The PfRd variants were made in a heterologous host M15 E. coli strain and had a total of 65 residues. The 12 extra residues come from N-terminus 6X histidine tag due to cloning in pQE30 vector. The protein sequence including N-terminus 6X histidine tag is given in the table below. All the mutants were purified by Ni-NTA affinity chromatography and the storage buffer was sodium phosphate buffer, 20 mM, pH 7.2.

### Aliphatic replacement mutations in PfRd

#### Materials used

The materials used were the same as described in the section on aromatic replacements. The forward and reverse primers used for PCR mutagenesis are described below. Primers for V4A, 2F-5′-GGATCCGCTAAATGGGCTTGCAAAATCTGTGG-3′, 2R-5′-CCACAGATTTTGCAAGCCCATTTAGCGG- 3′; Primers for I7A, 3F-5′-GCTAAATGGGTTTGCAAAGCTTGTGG-3′, 3R-5′- CGTAGATGTATCCACAAGCTTTGCAAACC- 3′; Primers for I11A, 5F-5′- GCAAAATCTGTGGATACGCTTACGACG-3′, 5R-5′-CCAGCGTCTTCGTCGTAAGCGTATCCAC-3′; Primers for I23A, 7F-5′-GGTGACCCTGACAATGGCGCTTCCCCG-3′, 7R-5′- GAATTTGGTACCCGGGGAAGCGCCATTG-3′; Primers for T27S, 8F-5′- CAATGGCATCTCCCCGGGTTCCAAATTCG-3′, 8R-5′-CAGTTCTTCGAATTTGGAACCCGG-3′; Primers for V37A, 11F-5′-CTGACGACTGGGCTTGTCCGATCTG-3′; Primers for 11R-5′- CACCACAGATCGGACAAGCCCAGTCG-3′; Primers for I40A, 12F-5′- GACGACTGGGTTTGTCCGGCTTGTGGTGC-3′, 12R-5′-GATTTTGGAGCACCACAAGCCGGACAAAC-3′

Mutations were done by PCR using QuickChange Site-Directed Mutagenesis Kit. Conditions and protocols were identical to those used for aromatic mutations (see previous sub-section). Mutants V16A, I19A and I23A were made by the same protocol.

The other four mutants were made by SOE PCR. Again, the final product obtained by the method was digested by BamH1 and HindIII enzymes and Quick Ligated into pQE30 digested previously by the same enzymes. The product was gel purified and transformed into XL1Blue E.coli Cells.

In all cases, PfRd and its mutants were made in a heterologous host M15 E. coli strain. The gene was cloned in the pQE30 vector. The encoded protein has a total of 65 residues. The 12 extra residues come from the N-terminal 6XHistag. The protein sequence is shown later in the paper, with colored characters pointing out the aliphatic and aromatic mutations.

## Results and Discussion

### Control experiments with affinity-tagged holo-PfRd and Apo-1 PfRd

We used a recombinant 6xHis (N-terminally) tagged form of PfRd produced in *Escherichia coli* for all experiments reported here. To examine whether the behavior of this tagged form of PfRd is identical to that of the untagged forms for which other authors have previously reported structural-biochemical or spectroscopic data, we first performed some control characterization experiments involving both tagged holo-PfRd and Apo-1 PfRd (produced through refolding of unfolded PfRd in the absence of iron). We also developed a method for the removal of the iron atom from folded holo-PfRd, to create and examine a different ‘apo’ form of PfRd (which we call Apo-2 PfRd). We then performed control experiments to verify whether this new apo form retains the folded structure of holo-PfRd at room temperature, in the absence of any denaturant. A summary of our control experiments (and results) is provided below.

**Figure 1:**
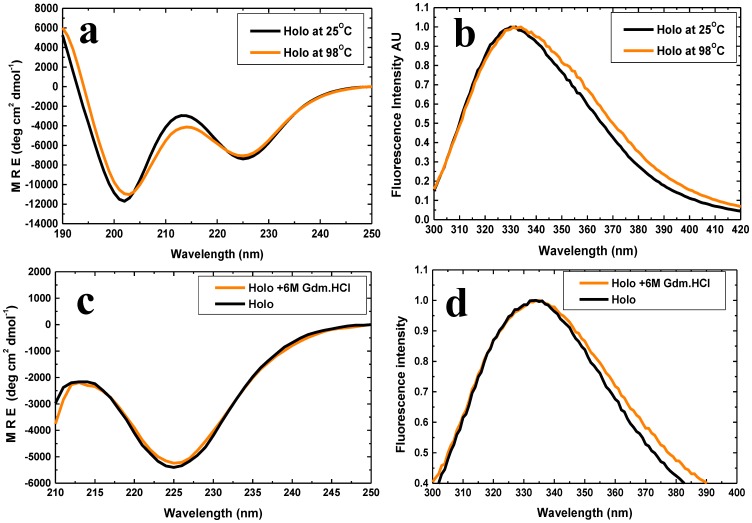
The effects of high temperature and denaturant (6 M Gdm.HCl) on the structure of (native) holo-PfRd. *Panel a*: Far-UV CD spectra at 25°C (black) and 98°C (orange). *Panel b*: Fluorescence emission spectra at 25°C (black) and 98°C (orange). *Panel c*: Far-UV CD spectra in the absence (black) and presence (orange) of 6 M Gdm.HCl, at room temperature. *Panel d*: Fluorescence emission spectra in the absence (black) and presence (orange) of 6 M Gdm.HCl, at room temperature.

**Figure 2:**
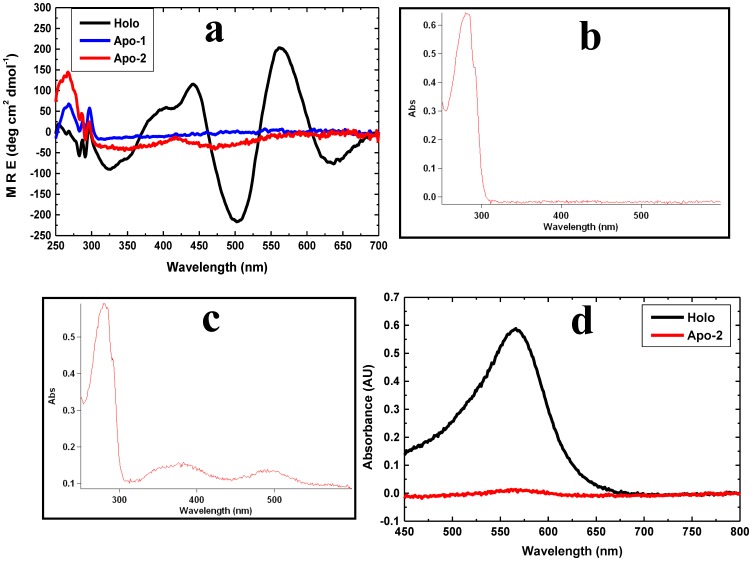
Status of the iron-sulfur cluster in Apo-1, Apo-2 and holo-PfRd. *Panel a*: Near-UV CD spectra of (native) holo-PfRd (black), Apo-1 PfRd (blue) and Apo-2 PfRd (red), showing the presence or absence of the iron-sulfur cluster-derived CD signatures between 300 nm and 700 nm. The features below 300 nm owe to other structural features (principally the aromatic residues). *Panel b*: Apo-2 PfRd's absorption spectrum, lacking the iron-sulfur cluster-derived absorption signatures at 390 nm and 490 nm. *Panel c*: Holo-PfRd's absorption spectrum showing iron-sulfur cluster-derived absorption signatures at 390 nm and 490 nm. *Panel d*: Apo-2 PfRd (red) releases no iron for uptake by ferrozine, following TCA treatment. In contrast, holo-PfRd releases iron (black), evident from the absorption band peaking at 560 nm, diagnostic of binding of ferrous iron by ferrozine. All released iron is reduced by beta-mercaptoethanol to ferrous form before ferrozine binding.

**Figure 3:**
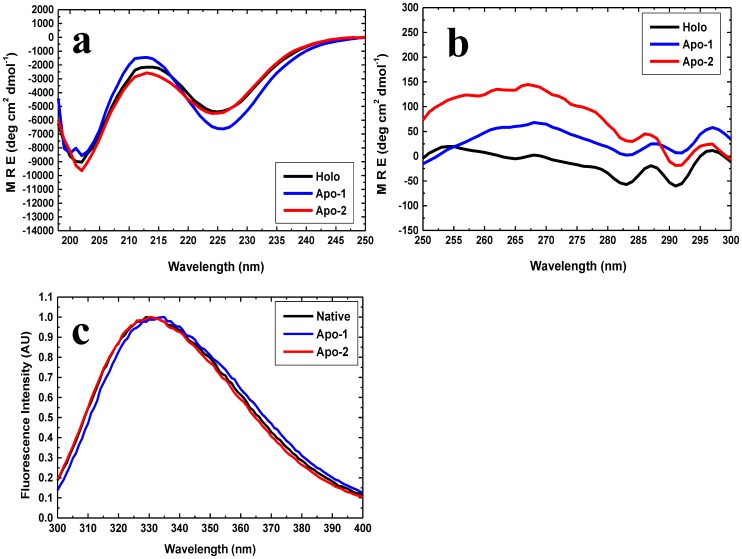
Similarity between the secondary and tertiary structural features of holo-PfRd, Apo-1 PfRd and Apo-2 PfRd. *Panel a*: Far-UV CD spectra of Apo-1 (blue), Apo-2 (red) and holo-PfRd (black). The spectrum has two bands. The 203 nm band owes to contributions from polyproline type II (PP-II) and random coil structures. The 225 nm band owes to contributions from secondary structures and aromatic residues. *Panel b*: Near-UV CD spectra of Apo-1 (blue), Apo-2 (red) and holo-PfRd (black). All bands owe to aromatic contributions. *Panel c*: Fluorescence emission spectra of Apo-1 (blue), Apo-2 (red) and holo-PfRd (black). The emission maximum of Apo-1 PfRd can be seen to be red-shifted by 2–3 nm.

**Figure 4:**
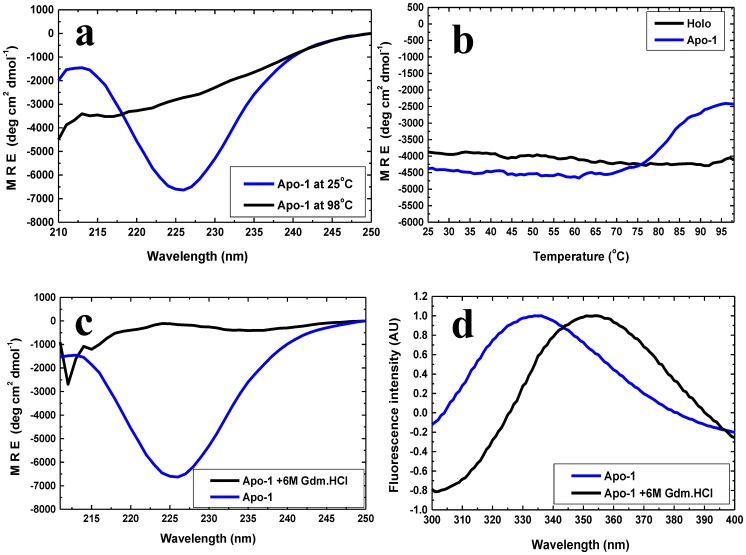
Poor stability of Apo-1 PfRd to denaturation by temperature and 6 M Gdm.HCl. *Panel a*: Far-UV CD spectra of Apo-1 PfRd at 25°C (blue) and 98°C (black), showing susceptibility to unfolding upon heating. Only the ∼225 nm band is shown. *Panel b*: Changes in mean residue ellipticity at 222 nm above the temperature of 70°C, shown by Apo-1 PfRd (blue) but not by holo-PfRd (black), as a function of heating. Apo-1 PfRd is lose over half of its CD signal strength at 222 nm. *Panel c* : Far-UV CD spectra of Apo-1 PfRd in the absence (blue) and presence (black) of 6 M Gdm.HCl. The spectra establish that Apo-1 PfRd loses structure completely (to a greater degree than is achieved by heating) in the presence of the denaturant. *Panel d*: Emission spectrum of Apo-1 PfRd (blue) shows a profound red shift in the presence of 6 M Gdm.HCl (black), from 335 nm to 355 nm.

Briefly, we found with the 6xHis tagged PfRd that: (i) native holo-PfRd is completely resistant to chemical denaturation at room temperature, and to thermal denaturation in the absence of denaturants [Figures 1a–d], as previously reported [8]; (ii) both Apo-1 PfRd and Apo-2 PfRd lack the characteristic spectral features of the iron-sulphur cluster seen in holo-PfRd, indicating that both are ‘apo’ forms of PfRd [Figures 2a–c]; (iii) Apo-2 PfRd releases no iron upon being unfolded, unlike holo-PfRd, which releases detectable iron, thus fully and incontrovertibly establishing that Apo-2 PfRd lacks iron [Figure 2d]; (iv) folded forms of Apo-1 PfRd, Apo-2 PfRd and holo-PfRd, in the absence of denaturant, display nearly overlapping far-UV CD spectra as well as very similar near-UV CD spectra and fluorescence emission spectra at room temperature [Figures 3a–c], as reported previously for Apo-1 PfRd and holo-PfRd [8], establishing that the three forms of PfRd have entirely similar secondary structural contents as well as grossly similar tertiary structural features (i.e., similar – if not identical - overall dispositions of aromatic residues, in terms of chiral environments and burial of side chains from the aqueous solvent); holo-PfRd displays an emission band with a wavelength maximum of emission (^em^λ_max_) of ∼333 nm, which shows no change in ^em^λ_max_ of PfRd in the presence of 6 M Gdm.HCl; (v) Apo-1 PfRd is poorly structural stable to chemical denaturation, as well as thermal denaturation, in comparison to holo-PfRd, as reported previously [Figures 4a–d].

It may be noted that the far-UV CD spectrum of holo-PfRd displays two band minima at 203 nm, and 225 nm, respectively. The 203 nm band owes to a combination of random coil and polyproline type II (PPII) structure. On the other hand, the 225 nm band owes to a combination of beta sheet secondary structure and also contributions from the excited stated induced CD transitions of the protein's 6 aromatic residues (W3, Y10, Y12, F29, W36, F48) which cluster together within the protein's core. The 203 nm band does not display much of a response to changes in the protein's environment (e.g., heat, or denaturant); however, changes owing to heat or denaturant appear to be reflected in changes in the intensity of the 225 nm band owing to changes in the protein's secondary structure (and three-dimensional structure, leading to changes in the organization of the aromatic cluster). Therefore, in the bulk of the remaining CD spectra shown in this paper, we display only changes in the 225 nm band. By way of representative example, in Figure 4c, we show only the 225 nm band in PfRd's far-UV spectrum, whereas in Figure 1a we have shown both bands.

### Extreme (holo-PfRd-like) resistance of Apo-2 PfRd to thermal and chemical denaturation

**Figure 5:**
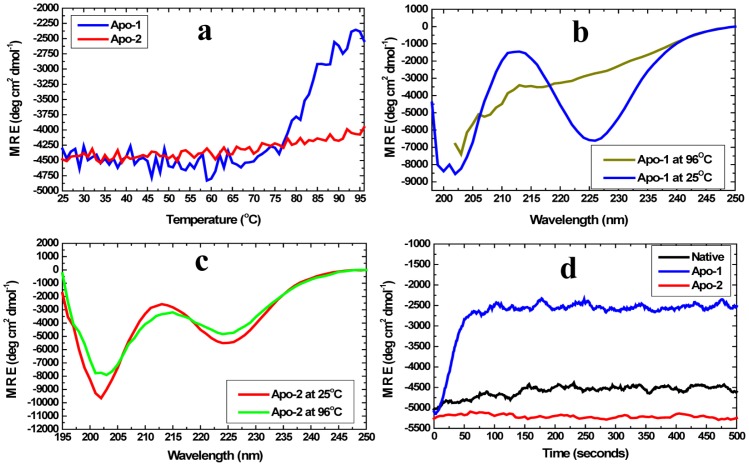
Apo-1 PfRd is thermolabile while Apo-2 PfRd is hyper-thermostable. *Panel a*. Changes in far-UV CD signal strength of the two proteins at 222 nm as a function of temperature. *Panel b*. Far-UV CD spectra of Apo-1 PfRd at two different temperatures. *Panel C*. Far-UV CD spectra of Apo-2 PfRd at two different temperatures. *Panel D*. Changes in far-UV CD signal strength of the two proteins, and of holo-PfRd (native) at 222 nm as a function of duration of heating at 95°C.

Figure 5a plots the strength of the CD mean residue ellipticity signal of Apo-2 PfRd at 222 nm as a function of temperature from 25°C to 98°C, alongside the comparable (unsmoothed) data for Apo-1 PfRd, demonstrating that like holo-PfRd - and unlike Apo-1 PfRd - Apo-2 PfRd undergoes no partial structure melting upon heating over this range of temperatures. The far-UV CD spectra of Apo-1 PfRd and Apo-2 PfRd at 25 and 98°C, respectively, are shown in Figures 5b and 5c, respectively, establishing that there are profound changes in the structure of Apo-1 PfRd at 98°C relative to its structure at 25°C. In contrast, no comparable changes are seen in the spectra for Apo-2 PfRd under identical heating conditions. Comparing this with the control spectra for holo-PfRd at these temperatures (Figure 1a), it becomes evident that Apo-2 PfRd and holo-PfRd are comparably thermo-stable, i.e., neither form displays any changes in its far-UV CD spectra upon heating. These conclusions are reinforced by the kinetic thermal structural perturbation data presented in Figure 5d, in which time course measurements of Apo-1 PfRd, Apo-PfRd and holo-PfRd, are contrasted as a function of the duration of incubation of each protein at a temperature of 96°C. Apo-1 PfRD undergoes significant partial unfolding within ∼50 seconds, whereas both Apo-2 PfRd and holo-PfRd remain fully-folded even after 3600 seconds (the figure shows data only up to 500 seconds).Therefore, Apo-1 PfRd is established to be thermo-labile in relation to both Apo-2 PfRd and holo-PfRd which are hyper-thermostable. This is especially intriguing in light of the fact that there is comparable (secondary) structural content in all three forms of PfRd.

**Figure 6:**
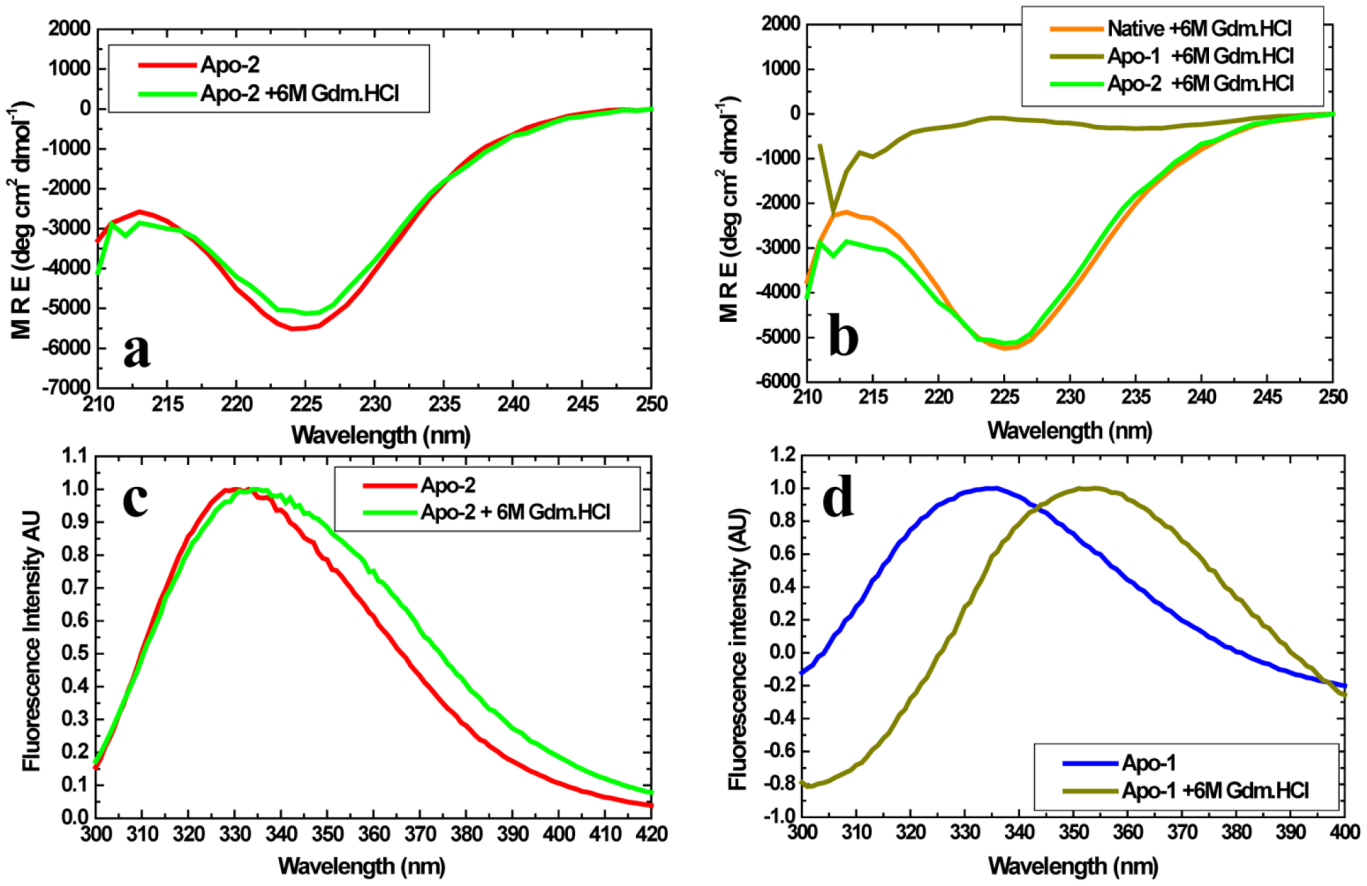
Apo-1 PfRd is labile to chemical denaturation, while Apo-2 PfRd resists chemical denaturation. *Panel a*. Far-UV CD spectra of Apo-1 PfRd at two different concentrations of Gdm.HCl. *Panel b*. Far-UV CD spectra of Apo-1 PfRd, Apo-2 PfRd and holo-PfRd (native) in 6M Gdm.HCl. *Panel c*. Fluorescence emission spectra of Apo-2 PfRd at two different concentrations of Gdm.HCl. *Panel d*. Fluorescence emission spectra of Apo-1 PfRd at two different concentrations of Gdm.HCl.

Similarly, Apo-2 PfRd shows extraordinary resistance to chemical denaturation, like holo-PfRd and unlike Apo-1 PfRd. Figure 6a presents far UV CD spectra of Apo-2 PfRd in the absence and presence of 6 M Gdm.HCl, following overnight incubation in the denaturant. The lack of any change in the spectral data demonstrates that there is no structural change whatsoever caused by the denaturant. Control spectra for Apo-1, and holo-PfRd, are shown in Figure 1c. It is clear from this data that holo-PfRd displays no changes in far-UV CD spectra in 6 M Gdm.HCl, whereas Apo-1 PfRd is completely denatured (Figure 6b).

Supporting data for the above circular dichroism-based observations is obtained from the fluorescence emission spectra of Apo-2 PfRd in the absence, and presence, of 6 M Gdm.HCl, as shown in Figure 6c. These spectra reveal that there is a very minor change in the emission spectral shape (a slight increase in bandwidth) without any significant alteration of Apo-2 PfRd's wavelength of maximal fluorescence emission (^em^λ_max_) in the presence of the 6 M Gdm.HCl. This behavior may be contrasted to Apo-1 PfRd's behavior in the presence of an equivalent concentration of the denaturant, as shown in Figure 6d. In Apo-1 PfRd, the ^em^λ_max_ is observed to shift to 353 nm in the presence of 6 M Gdm.HCl, establishing that Apo-1 PfRd undergoes sufficient unfolding for its tryptophan residues to become completely exposed to the solvent under these conditions. Therefore, Apo-2 PfRd is established to be as resistant to chemical denaturation as holo-PfRd, while Apo-1 PfRd is labile and vulnerable.

To exclude any possibility of there being any changes in the covalent chemical status of the iron-binding site following removal of iron (which could potentially give rise to alternative explanations for the observed extraordinary stability of Apo-2 PfRd), we performed alkylation experiments on Apo-2 PfRd and then used mass spectrometry to examine whether, upon removal of the iron atom, any disulphide bonds had been formed between Cys residues previously holding the iron atom. Figures S3a, S3b and S3c in File S1 establish that none of the four cysteine residues responsible for binding of iron engage in any disulphide formation, following the departure of the iron atom. Extensive details of the method by which Apo-2 PfRd was generated are mentioned in the materials and methods section. Details of the mass spectrometric experiment and analyses are mentioned in the discussion of Figure S3 in File S1. It may be noted that we also performed similar control experiments with Apo-1 PfRd as well (data not shown), examining its mass for signs of any covalent chemical changes, and none were found. Similarly, Apo-1 PfRd, Apo-2 PfRd and holo-PfRd were all chromatographed using gel filtration (data not shown) and found to display identical elution behavior, suggesting that they have identical hydrodynamic volumes. We would also like to mention that addition of iron to Apo-2 PfRd leads to no incorporation of iron into its structure. The same has been reported for Apo-1 PfRd [8], i.e., simple addition of iron does not lead to its incorporation. Both apo forms are folded sufficiently to be ‘closed’ to the entry of the iron atom at their iron-binding site. However, in the case of Apo-1 PfRd, it has been reported that refolding of unfolded PfRd molecules in the presence of iron does restore some of the unique absorption and CD spectroscopic characteristics associated with the oxidized iron atom in holo-PfRd's structure [8]. It may be noted though that no report exists about either the yield with which such iron incorporation occurs in refolding PfRd molecules, or even about whether holo-PfRd regenerated through such ‘iron-priming’ tends to be as stable as holo-PfRd formed through folding during biological synthesis *in vivo*. We attempted such iron-priming and found the yields to be very, very low. Also, we could not separate and purify trace amounts of iron-primed PfRd away from iron-lacking (refolded) PfRd to examine issues of structural stability.

### NMR spectroscopic comparison of the conformations of Apo-1 PfRd, Apo-2 PfRd and holo-PfRd

**Figure 7:**
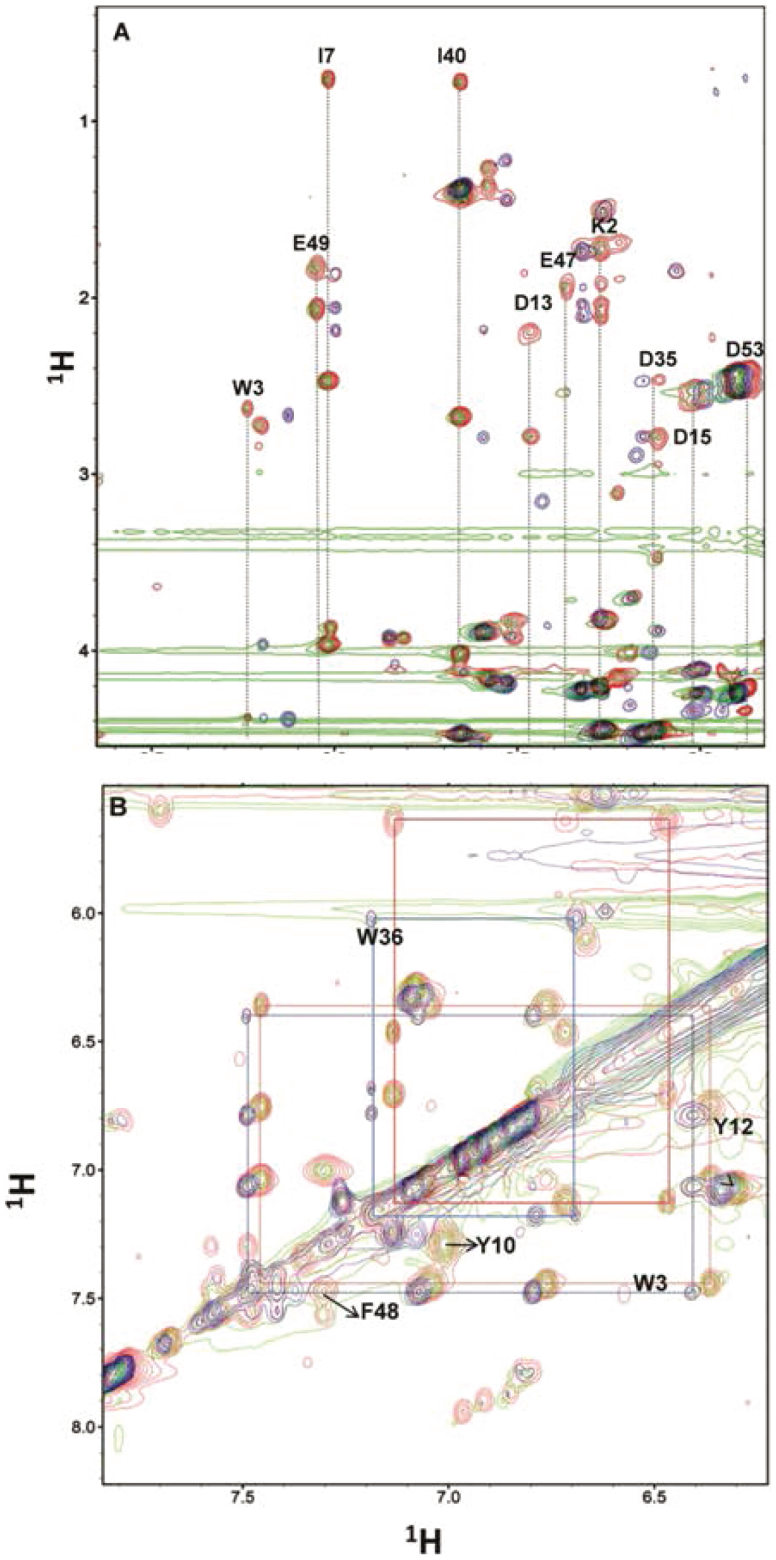
2D-1H TOCSY spectra for holo-PfRd (Red), Apo-1 PfRd (blue) and Apo-2 PfRd (green). *Panel A*. The amide region (finger print region) displaying some residues. Dotted lines indicate the spin systems. *Panel b*. Aromatic region in the TOCSY spectrum displaying peaks for the side chains of Trp 3, Tyr 10, Tyr 12, Trp 36, and Phe 48. TOCSY cross peaks connecting HZ2, HH2, HZ3 and HE3 are colored red for holo-PfRd and blue for Apo-1. The TOCSY mixing time used for the experiments was 60 msec.

Chemical shift assignments of holo-rubredoxin were carried out based on the BMRB entry 5601. Residues numbers in this entry differ from ours by one residue, i.e., every number in the BMRB entry is one higher than ours, according to our protein sequence. So, the residue numbers for the assignments in the figures presented below have been suitably corrected. As illustrated in Figure 7a, the majority of backbone amide chemical shifts appear to be conserved between holo-PfRd and Apo-2 PfRd. In contrast, significant differences are observed between holo-PfRd and Apo-1 PfRd. As backbone amide chemical shift changes are hallmarks of conformational change, it seems probable that the backbone conformation of Apo-1 PfRd is considerably different from that of holo-PfRd, whereas Apo-2 PfRd and holo-PfRd share very similar backbone conformations despite the absence of the bound Fe^3+^ ion in the former. The residues displaying marked changes in their backbone amide chemical shifts in Apo-1 PfRd are labeled in Figure 7a; these include Lys2, Trp3, Asp13, Asp35, Lys47, Glu49 etc. In the structure of PfRd, PDB ID 4AR6, these residues are scattered throughout the length of the protein and not restricted to any one particular region; however, it is clear that they are immediate neighbors of the aromatic residues Trp3, Tyr12, Trp36, and Phe48. This suggests that there is change in the backbone conformation of Apo-1 PfRd, relative to holo-PfRd, in several regions of the structure proximal to those hosting the molecule's aromatics.

Interestingly, differences in chemical shifts between holo-PfRd and Apo-1 PfRd are not limited only to the backbone, but also seen with the side chains. Both aliphatic and aromatic protons display chemical shift differences between Apo-1 PfRd and holo-PfRd. In contrast, the side chain resonances are almost fully conserved between holo-PfRd and Apo-2 PfRd. As shown in Figure 7b, the side chain chemical shifts for Trp3, Tyr10, Tyr12, Trp36 and Phe48are very similar for holo-PfRd and Apo-2 PfRd, whereas changes are observed in the case of Apo-1PfRd with regard to all of these residues. The maximum change in chemical shifts is seen with the Trp36 side chain in Apo-1 PfRd as shown in Figure 7b. The Trp3 side chain displays much less chemical shift change than the Trp36 side chain. Based on the ^1^H TOCSY data, one could conclude that the backbone and side chain conformations of Apo-2 PfRd are similar to those of holo-PfRd, whereas the backbone and side chain conformations of Apo-1 PfRd are substantively different in regions of the protein proximal to the aromatics, but otherwise reasonably similar to holo-PfRd and Apo-2 PfRd.

**Figure 8:**
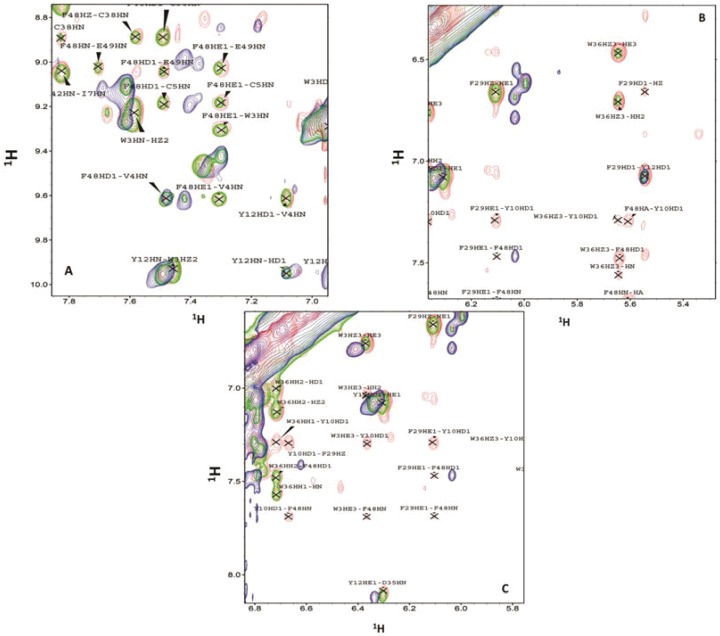
2D 1H NOESY spectra for holo-PfRd (Red), Apo-1 PfRd (blue) and Apo-2 PfRd (green) in the aromatic region. NOE associated with Phe 48 and Tyr 12 (*Panel A*) Phe 29 and Trp 36 (*Panel b*) and Trp 36, Trp 3 and Phe 29 side chains (*Panel c*). A NOESY mixing time of 150 msec was used in each experiment.

Figure 8 displays an overlay of the NOE's associated with aromatic side chains for holo-PfRd (colored red), Apo-1 PrRd (colored blue) and Apo-2 PfRd (colored green). Unlike the TOCSY spectra, the NOESY spectra for Apo-2 PfRD displays some differences in NOEs with holo-PfRd in the aromatic region as well, although most NOEs are similar. Backbone to sidechain NOEs are essentially the same between the two forms (data not shown). As shown in Figure 8a, two aromatic residues, namely Phe48 and Tyr12 display long range NOEs that are fully conserved between holo-PfRd and Apo-2 PfRd. These include NOEs between the side chain of Phe48 and backbone amides of Trp3, Val4, Cys5, Cys38, Glu47, Glu49 and the side chain of Tyr12 to the amide of Val4 and Asp35. Apart from NOEs involving these two aromatics, a few more NOEs are also conserved between the two forms, these being W36 HH2/Phe48 HD1, Phe29 HD11/Y12 HD1, Y10 HD1/V4 HN, and these are shown in Figures 8b and 8c. Some of the long range NOEs involving Phe29, Trp3, Trp36, Tyr10, however, are not observed in Apo-2 PfRd. These include Phe29 HE1/Tyr10 HD1, Phe29 HE1/Phe48 HD1, Phe29 HE1/Phe48 HN, Tyr10 HD1/Phe29 HZ, Tyr10 HD1/Phe48 HN, Trp3 HE3/Tyr10 HD1, Trp3 HE3/Phe48 HN, Trp36 HZ3/Phe48 HD1 etc. From the NMR data it emerges that the precise orientation of the Trp36 side chain with respect to the Phe48 side chain, and the Phe29 side chain to Tyr12 side chain, as well as the orientation of the Phe48 and Tyr12side chains with respect to the nearby amides are important in holding together the hydrophobic core up to a very high temperature. In contrast to the situation with Apo-2 PfRd where only some differences with holo-PfRd are observed, with the Apo-1 PfRd sample, NOE patterns are profoundly different from both holo-PfRd and Apo-2 PfRd, with virtually no similar NOEs. This potentially explains the observed low stability of Apo-1 PfRd, and the holo-PfRd-like stability of Apo-2 PfRd.

In summary, from the comparison of the NOESY data for the three forms, our conclusion is that the orientations of four aromatic side chains, W36, F48, F29 and Y12, with respect to each other are most important for retention of high stability, in terms of correlations between conserved orientations and high stability. Apart from this, the other key insight is that holo-PfRd's orientations of Phe 48 and Tyr 12 with respect to the nearby amides are also retained in Apo-2PfRd, but not in Apo-1 PfRd. The similarities between the NMR spectra of Apo-2 PfRd and holo-PfRd are satisfying, in that they show that Apo-2 PfRd is not profoundly altered by iron removal, i.e., there is no profound conformational transition to an Apo-1 like structure upon the removal of iron, establishing that the structure formed after binding of iron is kinetically-stable. Structures within such kinetic traps display extraordinary stabilities which are not true thermodynamic stabilities, but merely apparent stabilities, owing to the slowing down of unfolding by the high energy barriers ‘fencing-in’ the structure. This study of Apo-2 PfRd, holo-PfRd, and Apo-1 PfRd thus assumes special significance because of the clear correlations it suggests between differences in structure and apparent stability. Considering the following facts together, namely : (i) that the NMR spectra of Apo-2 PfRd and holo-PfRd are much more alike, while that of Apo-1 PfRd is substantial different, with most of the differences locating to the aromatic residues and their mutual orientation and packing, and (ii) that Apo-1 PfRd is unfolded rapidly by high temperature and chemical denaturants, whereas Apo-2 PfRd is not unfolded, it appears that there could be a cause-effect relationship between the differential aromatic residue packing and the differences in the structural stabilities of Apo-1 PfRd and Apo-2 PfRd.

**Figure 9:**
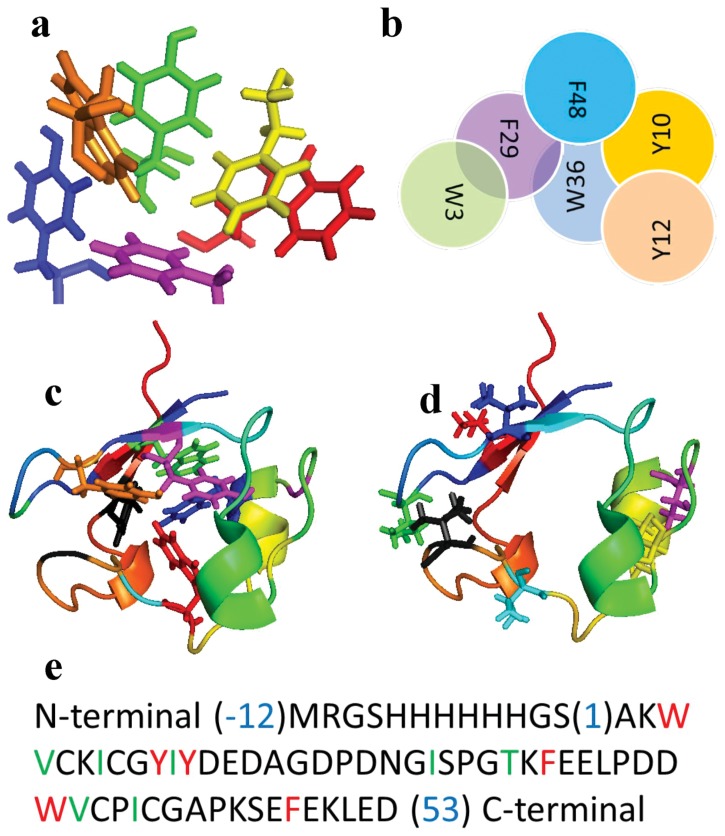
*Panel a*. The aromatic residue cluster of holo-PfRd. Residues shown are W3 (red), Y10 (blue), Y12 (green), F29 (yellow), W36 (orange), F48 (magenta). *Panel b*. A schematic representation of the aromatic cluster in holo-PfRd, showing interactions between different aromatic residues. *Panel C*. The six aromatic residues in the core of holo-PfRD which were mutationally substituted by alanine, shown within the structural context of the polypeptide backbone of the protein. *Panel D*. The seven aliphatic residues in the periphery of holo-PfRD which were mutationally substituted by alanine, or serine, shown within the structural context of the polypeptide backbone of the protein. *Panel E*. The sequence of N-terminally 6XHis tagged holo-PfRd. Aromatic residues subjected to substitution mutagenesis are shown in red; Aliphatic residues subjected to substitution mutagenesis are shown in green/blue.

Interestingly, a relatively recent theoretical analysis of PfRd's extreme stability (based purely on quantum chemical considerations) posits that the aromatic residue cluster lying within the core of PfRd's structure probably plays an important role is stabilizing the protein [12]. What is interesting about our experimental data is the hint that it is not the presence of an aromatic cluster in the PfRd molecule *per* se which is of significance to its stability, but rather the details of the packing and orientations of the aromatics in the cluster which are of significance. What is also interesting is that these aromatics can pack so differently in molecules that are identical from the view of covalent chemical structure (confirmed through mass spectrometry; data not shown) and quaternary structure (confirmed through gel filtration studies; data not shown). Significantly, the exact set of residues identified by us as being subtly differentially packed in Apo-1 PfRd and in Apo-2 or holo-PfRd – i.e., consisting of the same aromatics residues and flanking aliphatic residues (with sequence numbering offset by 1) - has been previously identified to constitute the core of PfRd, displaying the slowest H-D exchanges in NMR experiments comparing PfRd and mesophile rubredoxins [14]. The structure of the cluster is shown in Figure 9a and the cluster is also shown schematically in Figure 9b in terms of the numbers and natures of aromatic-aromatic interactions, with further details of the structure and geometry of these interactions presented in the Supporting results and discussion and Figures S4a–S4g in File S1. Additionally, Figures 9c and 9d visually show that all the aromatic residues of PfRd lie within its hydrophobic core whereas a large proportion of the protein's aliphatic residues lie on its surface. The positions of the aliphatic and aromatic residues in PfRd's sequence are shown in Figure 9e.

The rationale and analyses underlying the particular schematic shown in Figure 9b are presented in the discussion section accompanying Figures S4a–g in File S1. Briefly, in this schematic figure, it is shown that three of the six aromatic residues in PfRd engage in extensive and intimate interactions with other aromatic residues. These three residues are F29, W36 and F48. Of these, residue F29 interacts with both W3 and F48, acting like a bridge between these two residues. Likewise, residue W36 interacts with four other aromatic residues, F29, F48, Y10 and Y12, acting like a bridge between F29 and Y12. Residue F48 interacts intimately with residues F29, W36 and Y10; interestingly, this residue, i.e., F48, also acts as a bridge between F29 and Y10, but it is not the only bridge between these residues because F29 and Y10 are also bridged by W36.

### Substitution of key aromatics: Predictions of effects of alanine substitutions

Based on the schematic shown in Figure 9b and the detailed analyses presented above, we are inclined to predict that replacement of either of the two residues, F29, or W36, by alanine would destroy significant portions of the aromatic cluster in PfRd's core. F29 acts as the only bridging aromatic residue between W3 and F48. Likewise, W36 acts as the only bridging residue between F29 and Y12. Therefore, these two alanine substitutions could be predicted to have profound effects on the stability of PfRd, and perhaps also effects on structure formation in PfRd. With the third ‘bridge’ residue, F48, however, we would not anticipate a similarly profound effect of making an alanine substitution, since W36 also bridges the two residues bridged by F48, i.e., F29 and Y10. To test these predictions, we decided to replace all six of PfRd's aromatic residues by alanine. As control experiments, we also replaced a significant number of aliphatic residues by alanine (and, in one case, for T27, by serine). The results obtained experimentally by making these substitutions are described below.

### Substitution of key aromatics by alanine: Profound effects on holo-PfRd's structure and stability

**Figure 10:**
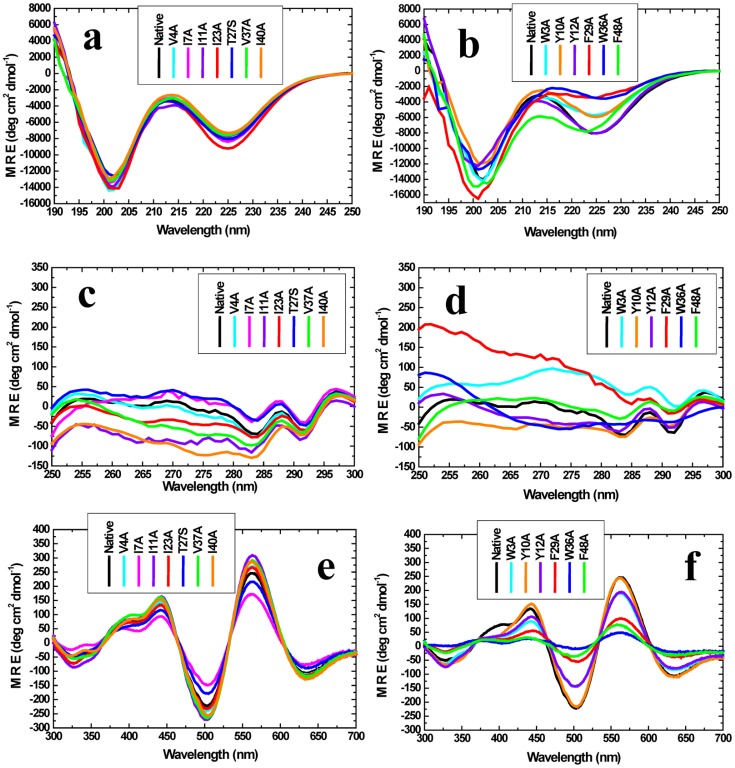
*Panel a.* Far-UV CD spectra of holo-PfRd (native) and different *aliphatic* residue substitution mutants. *Panel b*. Far-UV CD spectra of holo-PfRd (native) and different *aromatic* residue substitution mutants. *Panel C*. Near-UV aromatic CD spectra of holo-PfRd (native) and different *aliphatic* residue substitution mutants. *Panel D*. Near-UV aromatic CD spectra of holo-PfRd (native) and different *aromatic* residue substitution mutants. *Panel E*. Near-UV iron-sulphur cluster CD spectra of holo-PfRd (native) and different aliphatic residue substitution mutants. *Panel F*. Near-UV iron-sulphur cluster CD spectra of holo-PfRd (native) and different aliphatic residue substitution mutants.

The data from the structural-biochemical examination of mutants is shown in Figure 10 for alanine replacement mutations of aromatic residues in PfRd's core, and for alanine/serine replacement mutations of aliphatic residues on PfRd's surface. The data in Figure 10a clearly demonstrates that seven different individual replacement mutations of aliphatic residues to alanine, or serine (V4A, I7A, I11A, I23A, T27S, V37A, I40A) elicit negligible changes in PfRd's structure. In contrast, as Figure 10b shows, four out of the six mutations of aromatic residues to alanine (W3A,Y10A, F29A, W36A) result in profound changes in PfRd's far-UV CD spectrum, while a fifth mutation (F48A) elicits less profound but very significant changes. As explained already, the far-UV CD spectrum of PfRd and, in particular, the ∼225 nm negative band in the CD spectrum, results from contributions from both secondary structural elements and aromatic residues. The ∼203 nm band owes to contributions from random coil and PP-II structures. In Figures 10a and 10b, changes are seen mainly in the ∼225 nm band, and also in the ∼203 nm band where aromatic substitution mutations are made, while almost no changes whatsoever are see in either band in cases involving aliphatic residue substitutions. Therefore, the differences obtained in the ∼225 nm and ∼203 nm bands upon individual aromatic mutations to alanine represent alterations in the formation and packing of the aromatic cluster, accompanied by some alterations in the structure of the protein as a whole. Figures 10c and 10d show that the main spectral features of the near-UV CD spectra are more conserved in the case of the aliphatic residue substitutions, while in the case of the aromatic residue substitutions there is a profound effect (a flattening) of the spectrum in the W36A mutant, with less profound effects seen with the other substitution mutants. Based on this observation, it appears likely that the two positive bands seen at ∼287 and ∼295 nm owe more to W36 than to W3. Likewise, there is a flattening seen in the spectrum in the region of the positive band observed in PfRd at ∼255 nm in the case of the F48A mutation, suggesting that F48 is more responsible for this band than F29.

Notably, in the case of all these substitution mutations, there was no ‘absolute’ effect of the mutations on iron binding. All of the mutants were red in color when they were purified, with some differences in the intensity of the color per unit concentration of protein (as would be expected to occur, if there were differences in the environment of the oxidized iron atom). The binding of iron by all mutants is also evident from Figure 10e and 10f, in which it is seen that each mutant shows all the characteristic spectral features of holo-PfRd in the entire range of wavelengths from 300 to 700 nm, with only quantitative differences amongst mutants.

This demonstrates that the binding and retention of iron is an independent event which is not contingent on the formation of the correct folded structure with the correct packing of aromatic residues in PfRd's core; rather, iron binding would appear to be an early event during folding, with the potential of guiding the folding of the chain to the correct structure when all aromatic residues are present and available to pack in the native fold. When these residues are not available (as happens to be the case with the mutants), the correct native structure does not fully form (evident from the spectra in Figures 10a and 10b), even though there is initial binding of iron (evident from the spectra in Figures 10e and 10f). Conversely, when iron is not available to be bound, the aromatic residues do form an aromatic cluster, but this cluster is formed using a different packing geometry from that which applies when iron is available, and bound, early during folding. Either way, it becomes evident that the nature of the aromatic residue cluster is critical in determining whether the correct native structure has formed in PfRd and also whether the folded structure is ordinarily thermostable (as in Apo-1 PfRd), or extraordinarily thermostable (as in holo-PfRd, and also in Apo-2 PfRd in which the structure was allowed to form before iron was removed). We also examined the effects of heating of the mutants as a function of time in terms of changes in their mean residue ellipticity signals, as shown in Figures S5a–S5f in File S1. Different mutants behave differently, with some showing a decrease in the signal and other showing an increase, but the overall data concerning the individual stabilities of the mutants is in consonance with the conclusions and interpretations presented above and in Figures 10a–10f.

Collectively, the data suggests that iron-binding determines what kind of structure is ultimately formed, with which kind of residue packing of aromatics in the protein's core. The iron-dependent determination of residue packing (in particular, aromatic residue packing) thus determines the protein's hyperthermal stability, rather than the presence of iron in PfRd's iron-binding site. This is especially significant, given the wealth of information that is already available regarding aromatic clusters and the role that they could potentially play in stabilizing proteins [15], [16].

## Conclusions

**Figure 11:**
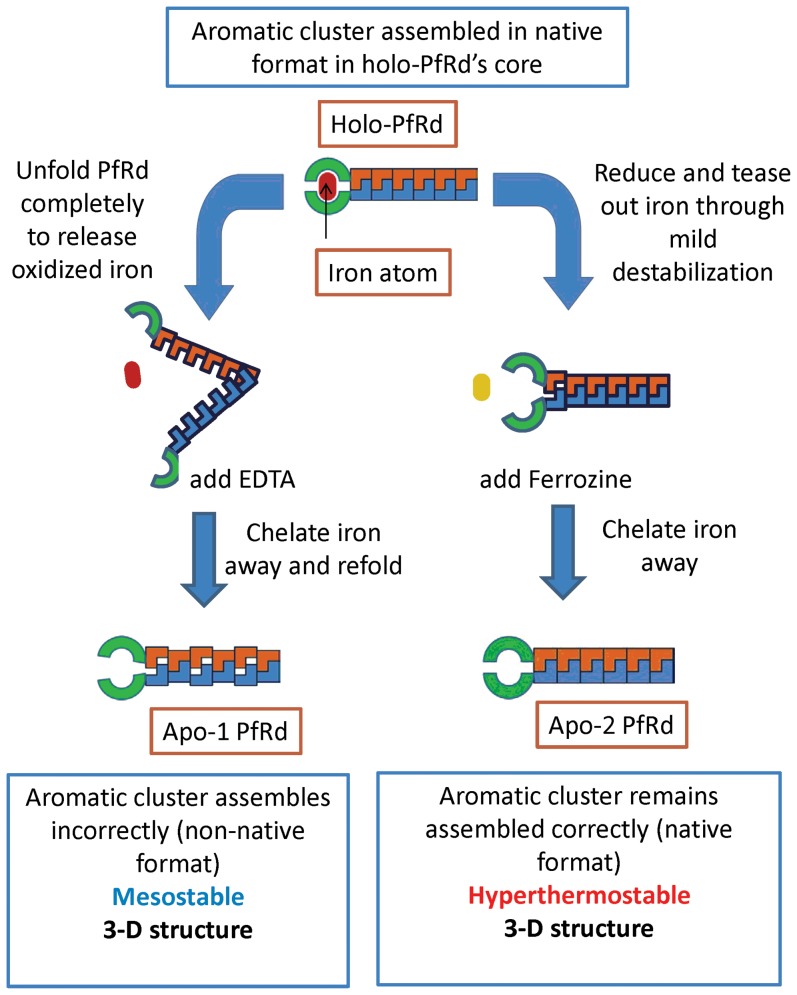
A schematic figure summarizing key conclusions.

We have effectively demonstrated that two iron-lacking ‘apo’ forms of PfRd, namely Apo-1 PfRd and Apo-2 PfRd, differ profoundly in their structural-biochemical and stability characteristics. While Apo-1 PfRd lacks PfRd's extreme structural stability, Apo-2 PfRd possesses it. Both Apo-1PfRd (made by unfolding PfRd and refolding it in the absence of iron), and Apo-2 PfRd (made by removing iron from partially-destabilized PfRd) have conformations that are virtually identical to holo-PfRd. Indeed, the conformations of the three forms are virtually indistinguishable, based on CD and fluorescence spectral features. However, NMR spectroscopy suggests that while aliphatic residues in the three forms are largely similarly packed and organized, most aromatic residues are not. The packing of aromatic residues in Apo-2 PfRd has more in common with holo-PfRd than either of these forms has with Apo-1 PfRd. Studies of mutants made by substituting six aromatic residues with alanine, and seven aliphatic residues with alanine, or serine, show that all of these mutants bind iron, even though a number of them (i.e., some of the aromatic substitution mutants) fail to form native-like aromatic clusters, clearly indicating that iron-binding is not contingent on the packing of the aromatic cluster, whereas packing of the aromatic cluster is clearly contingent on iron-binding. These conclusions are schematically represented in Figure 11 which summarizes the bulk of our findings, and hints at the cause for the observed difference in behavior being subtle conformational differences in the packing of side-chains in the molecule's core.

The *first (and most important) insight* that emerges from this result is the understanding that it is not the presence, or absence, of an iron atom which determines PfRd's extraordinary structural stability, since Apo-2 PfRd possesses such stability despite its lack of an iron atom. A related *second important insight* that emerges from this result is that it is possible for two forms of a single protein to adopt extremely similar secondary and tertiary structural features and yet be entirely dissimilar in terms of their relative stabilities to denaturation. Another related, *third important insight* is the understanding that subtle differences in tertiary structural organization (i.e., in the details of packing of aromatic side-chains within a protein's structure) can make a very profound difference in determining the protein's stability. A *fourth important insight* that emerges is that while we confirm earlier observations suggesting that it is possible to refold PfRd's polypeptide chain to an almost PfRd-like conformation in the absence of iron, we show that this conformation differs from holo-PfRd and does not have the stability of holo-PfRd. We have attempted to denature and refold Apo-1 PfRd in the presence of iron and while we can get iron binding to occur, we cannot demonstrate that we obtain the extraordinarily stable folded conformation of holo-PfRd, as the yields of iron-containing refolded protein are very low and it is not possible to separate these molecules and study them yet. Thus, we establish that PfRd cannot truly be refolded quantitatively. Thus, PfRd will have to be taken off the list of hyperthermophile proteins that can be substantively refolded from unfolded state. The *fifth important insight* to emerge from these studies is that binding of a metal atom can occur independently of (and earlier than) the formation of the correct hydrophobic core, during folding; indeed, in PfRd's case, the binding of iron determines the formation of the correct hydrophobic core, constituted of an aromatic cluster. The *final important insight* is that this may be the first protein in which the existence as well as the details of packing and orientation of residues constituting an aromatic cluster are shown to profoundly affect the protein's structural stability, whereas aromatic clusters have previously been thought to contribute to protein stability based on theoretical analyses. A *further point of note* is that we have demonstrated, in work slated to appear concurrently in this journal (examining the cold-denaturation behavior of PfRd during thermo-chemical denaturation and subsequent to such denaturation), that the iron-sulphur cluster is protected from beta-mercaptoethanol by the side-chains that surround the cluster, requiring us to have to use mild denaturants to tease out the iron to create Apo-2 which possesses the stability of PfRd.

## Supporting Information

File S1 **Supporting information and figures.** **Figure S1,** *Panel* a : Control solutions lacking ferrozine. Tube-1 contains a ferric chloride solution. Tube-2 contains ferric chloride and beta-mercaptoethanol. Tube-3 contains ferric chloride, beta-mercaptoethanol and guanidium hydrochloride. *Panel* b : Sample solutions containing ferrozine. Tube-1 contains ferrozine added to ferric chloride. Tube-2 contains ferrozine added to ferric chloride pre-mixed with beta-mercaptoethanol. Tube-3 contains ferrozine added to ferric chloride pre-mixed with beta-mercaptoethanol and guanidium hydrochloride. Tube-4 contains ferrozine added to ferrous sulphate. **Figure S2,** *Panel a* : Elution of free ferrozine (∼17 ml and ∼20 ml) on a Superdex Peptide (GE) column in the absence of any iron or protein. *Panel b* : Fe^2+^-bound ferrozine (two eluting species at 12.5 ml and 14.0 ml) separated from free ferrozine (∼17 ml) on the same column. *Panel c* : Fe^2+^-bound ferrozine (∼12.5 ml and ∼14.0 ml) separated from free ferrozine (∼17 ml and 20 ml) and PfRd protein (∼10 ml) on the same column. *Panel d*: Elution of PfRd protein (∼10 ml) on a Superdex Peptide (GE) column in the absence of any ferrozine. **Figure S3,** *Panel a* : MALDI-TOF MS spectrum of Apo-2 PfRd (including the N-terminal 6xHis tag), showing that the protein has a mass of 7292 Da. The theoretically expected mass is ∼7294 Da. *Panel b* : MALDI-TOF MS spectrum of N-terminally 6xHis tagged Apo-2 PfRd alkylated by iodoacetic acid (IAA) after treatment with beta mercaptoethanol. The masses of 7534, and 7409 Da represent species carrying four, and two, IAA aductions, respectively, indicating that PfRd's four cysteine residues are free and available to be alkylated in the presence of beta-mercaptoethanol. *Panel c* : MALDI-TOF MS spectrum of N-terminally 6xHis tagged Apo-2 PfRd alkylated by iodoacetic acid (IAA) without any treatment with beta mercaptoethanol. The masses of ∼7523.95, ∼7462.27, ∼7406 and ∼7345 Da represent species carrying four, three, two, and one IAA aductions, respectively, with the mass peak with the highest intensity representing the population with all four of PfRs's cysteine residues modified. The molecule's cysteine residues are thus free and available to be alkylated (and not disulfide bonded). **Figure S4, Organization of the aromatic cluster in holo-PfRd, showing different aromatic interactions amongst the molecules six aromatic residues, namely W3 (green), Y10 (orange), Y12 (magenta), F29 (blue), W36 (red) and F48 (black).** **Figure S5,** *Panels a and b* : Changes in the CD MRE signal at 222 nm of aliphatic (Panel a) and aromatic (Panel b) substitution mutants as a function of increasing temperature, in the absence of denaturant. *Panels c and d* : Changes in the CD MRE signal at 222 nm of aliphatic (Panel c) and aromatic (Panel d) substitution mutants as a function of increasing temperature, in the presence of 6 M Gdm. HCl. *Panels e and f* : Time course of changes in the CD MRE signal at 222 nm of aliphatic (Panel e) and aromatic (Panel f) substitution mutants at 95 degrees Centigrade, in the presence of 6 M Gdm.HCl.
(PDF)
