# Supplementary material for: The Key to the Extraordinary Thermal Stability of P. furiosus Holo-Rubredoxin: Iron Binding-Guided Packing of a Core Aromatic Cluster Responsible for High Kinetic Stability of the Native Structure
Source: PLoS One. 2014 Mar 6;9(3):e89703. doi: 10.1371/journal.pone.0089703 (PMC3945938; doi:10.1371/journal.pone.0089703)
Supplement: File S1 — Supporting information and figures. Figure S1, Panel a : Control solutions lacking ferrozine. Tube-1 contains a ferric chloride solution. Tube-2 contains ferric chloride and beta-mercaptoethanol. Tube-3 contains ferric chloride, beta-mercaptoethanol and guanidium hydrochloride. Panel b : Sample solutions containing ferrozine. Tube-1 contains ferrozine added to ferric chloride. Tube-2 contains ferrozine added to ferric chloride pre-mixed with beta-mercaptoethanol. Tube-3 contains ferrozine added to ferric chloride pre-mixed with beta-mercaptoethanol and guanidium hydrochloride. Tube-4 contains ferrozine added to ferrous sulphate. Figure S2, Panel a : Elution of free ferrozine (∼17 ml and ∼20 ml) on a Superdex Peptide (GE) column in the absence of any iron or protein. Panel b : Fe2+-bound ferrozine (two eluting species at 12.5 ml and 14.0 ml) separated from free ferrozine (∼17 ml) on the same column. Panel c : Fe2+-bound ferrozine (∼12.5 ml and ∼14.0 ml) separated from free ferrozine (∼17 ml and 20 ml) and PfRd protein (∼10 ml) on the same column. Panel d: Elution of PfRd protein (∼10 ml) on a Superdex Peptide (GE) column in the absence of any ferrozine. Figure S3, Panel a : MALDI-TOF MS spectrum of Apo-2 PfRd (including the N-terminal 6xHis tag), showing that the protein has a mass of 7292 Da. The theoretically expected mass is ∼7294 Da. Panel b : MALDI-TOF MS spectrum of N-terminally 6xHis tagged Apo-2 PfRd alkylated by iodoacetic acid (IAA) after treatment with beta mercaptoethanol. The masses of 7534, and 7409 Da represent species carrying four, and two, IAA aductions, respectively, indicating that PfRd's four cysteine residues are free and available to be alkylated in the presence of beta-mercaptoethanol. Panel c : MALDI-TOF MS spectrum of N-terminally 6xHis tagged Apo-2 PfRd alkylated by iodoacetic acid (IAA) without any treatment with beta mercaptoethanol. The masses of ∼7523.95, ∼7462.27, ∼7406 and ∼7345 Da represent species carrying four, three, two, and one [file pone.0089703.s001.pdf]

Supplementary file

The key to the extraordinary thermal stability of *P. furiosus* holo-rubredoxin: Iron binding-guided packing of a core aromatic cluster responsible for high kinetic stability of the native structure

SatyaPrakash,[1,2] Monica Sundd,[3] Purnananda Guptasarma[1,2]

[1] Department of Biological Sciences, Indian Institute of Science Education and Research (IISER) Mohali, Knowledge City Sector-81, SAS Nagar, Manauli P.O., Punjab 140306, India

[2] Protein Engineering Division, Institute of Microbial Technology (IMTECH), Sector 39-A, Chandigarh 160036, India

[3]NMR Laboratory, National Institute of Immunology (NII), Aruna Asaf Ali Marg,
New Delhi - 110067, India

The interplay of reduced and oxidized iron with B-ME and ferrozine

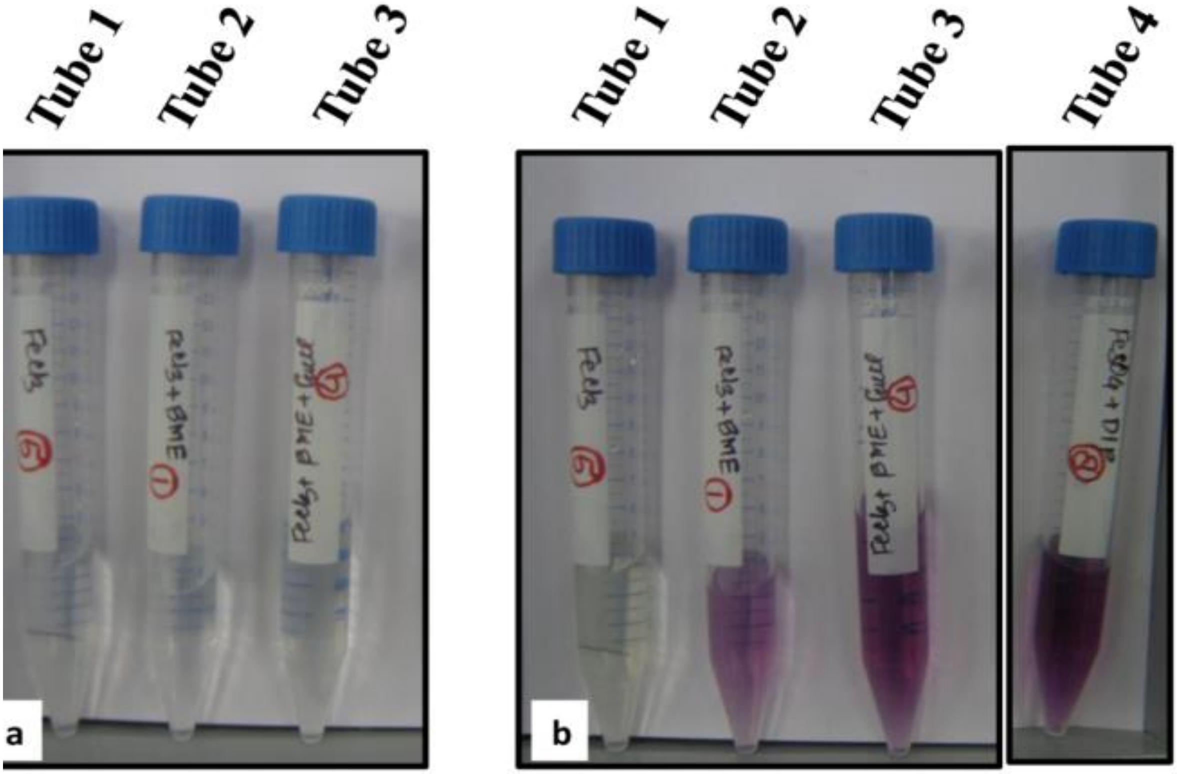

Figure S1 :

Panel a : Control solutions lacking ferrozine. Tube-1 contains a ferric chloride solution. Tube-2 contains ferric chloride and beta-mercaptoethanol. Tube-3 contains ferric chloride, beta-mercaptoethanol and guanidium hydrochloride. *Panel* b : Sample solutions containing ferrozine. Tube-1 contains ferrozine added to ferric chloride. Tube-2 contains ferrozine added to ferric chloride pre-mixed with beta-mercaptoethanol. Tube-3 contains ferrozine added to ferric chloride pre-mixed with beta-mercaptoethanol and guanidium hydrochloride. Tube-4 contains ferrozine added to ferrous sulphate.

DISCUSSION OF FIGURE S2

Use of β-ME-based iron reduction, ferrozine-binding and (lastly) gel filtration chromatography through ferrozine-absorption monitoring TO establish the method developed for generation of Apo-2 PfRd

**** Free ferrozine - Control.*** As control, a gel filtration (Superdex Peptide resin) chromatogram obtained with free ferrozine is shown in Fig. S2a, using an 'overloaded' amount of ferrozine [500 µl of 5 mM ferrozine; see materials and methods]. The traces in blue and red show the absorptions at 280 nm and 560 nm respectively. The free ferrozine can seen to show absolutely no absorption at 560 nm. On the other hand, the chromatogram does display the 280 nm absorption that it is expected to, on account of ferrozine's aromatic structure which facilitates absorption at 280 nm. Interestingly, free ferrozine is seen to elute as two separate species, at elution volumes of ~18.2 ml and ~19.67 ml. Of these, the ~19.67 ml elution constitutes only a small fraction of the total ferrozine population, with the bulk of the population eluting at ~18.2 ml.
**** Fe^{2+}-bound ferrozine - Control****.* Fig. S2b shows the chromatogram obtained with a 500µl sample loading of 0.139 mM ferrozine originally mixed with ferrous iron (final concentration 0.277 mM). A tiny amount of free trace ferrozine is seen to elute at ~18.2 ml, using 280 nm monitoring. This elution shows no absorption at 560 nm, confirming that it is free ferrozine. The ~19.67 ml elution is undetectable, presumably because it constitutes a minority population of free ferrozine as already established by the data in Fig. S2a. It is noteworthy that the bulk of the 560 nm absorption in the chromatogram (Fig. S2b) is seen in the form of two elutions at ~12.83 ml and ~14.38 ml. Interestingly, the ~12.83 ml elution contains a minor subspecies ~12.27 ml, which shows up as a shoulder associated with the ~12.27 ml peak. All three species absorb light at 560 as well as 280 nm, indicating that they all correspond to different forms of Fe^{2+}-bound ferrozine.
**** Separating Fe^{2+}-bound ferrozine and protein (Apo-2 PfRd)****.* Fig. S2c shows the chromatogram obtained with a 500 µl loading of the iron-containing protein, PfRd, which has been caused to release its iron, with the iron already having been substantively converted to the ferrous (Fe^{2+}) form by the addition of beta-mercaptoethanol. As already mentioned in Materials and Methods, 2-mercaptoethanol is known to reduce ferric iron to ferrous iron. The loaded solution had a final protein concentration of 1mg/ml (~0.139 mM protein) as well as ferrozine (also at ~0.139 mM). The first three peaks of iron bound ferrozine elute at almost the same positions as in the earlier chromatograms. The peaks of free unimolecular ferrozine and dimeric ferrozine (formed through ring-stacking interactions) are seen at ~19.56 ml and ~17.85 ml. A very broad peak adjacent to the ~19.56 ml peak which elutes up to an elution volume of 25 ml is of free iron and Gdm.HCl. The protein itself (i.e., iron-lacking PfRd, or Apo-2 PfRd) is seen to elute soon after the void volume of the column, at ~10.13 ml. The elution of the protein was confirmed by appropriate control experiments including chromatography of PfRd [Fig. S6d] and other experiments involving SDS-PAGE and MALDI-MS (data not shown). Fe^{2+}-bound ferrozine has a much bigger hydrodynamic volume than free ferrozine, because ferrozine forms trimers with bound ferrous iron. So, this species is seen to elute so much earlier than free ferrozine that baseline-resolution of Fe^{2+}-bound and free ferrozine is achieved.
**** Free protein (holo-PfRd) - Control****.* Fig. S2d shows the elution volume of holo-PfRd, as a control. It can be seen from this figure that the elution volume of ~10.13 ml seen for Apo-2PfRd is the expected elution volume of holo-PfRd (both forms are monomers).

So, the above chromatographic method was used to generate Apo-2 PfRd, with iron removed.
The Supporting figures referred to on this page are shown on the next page with legends

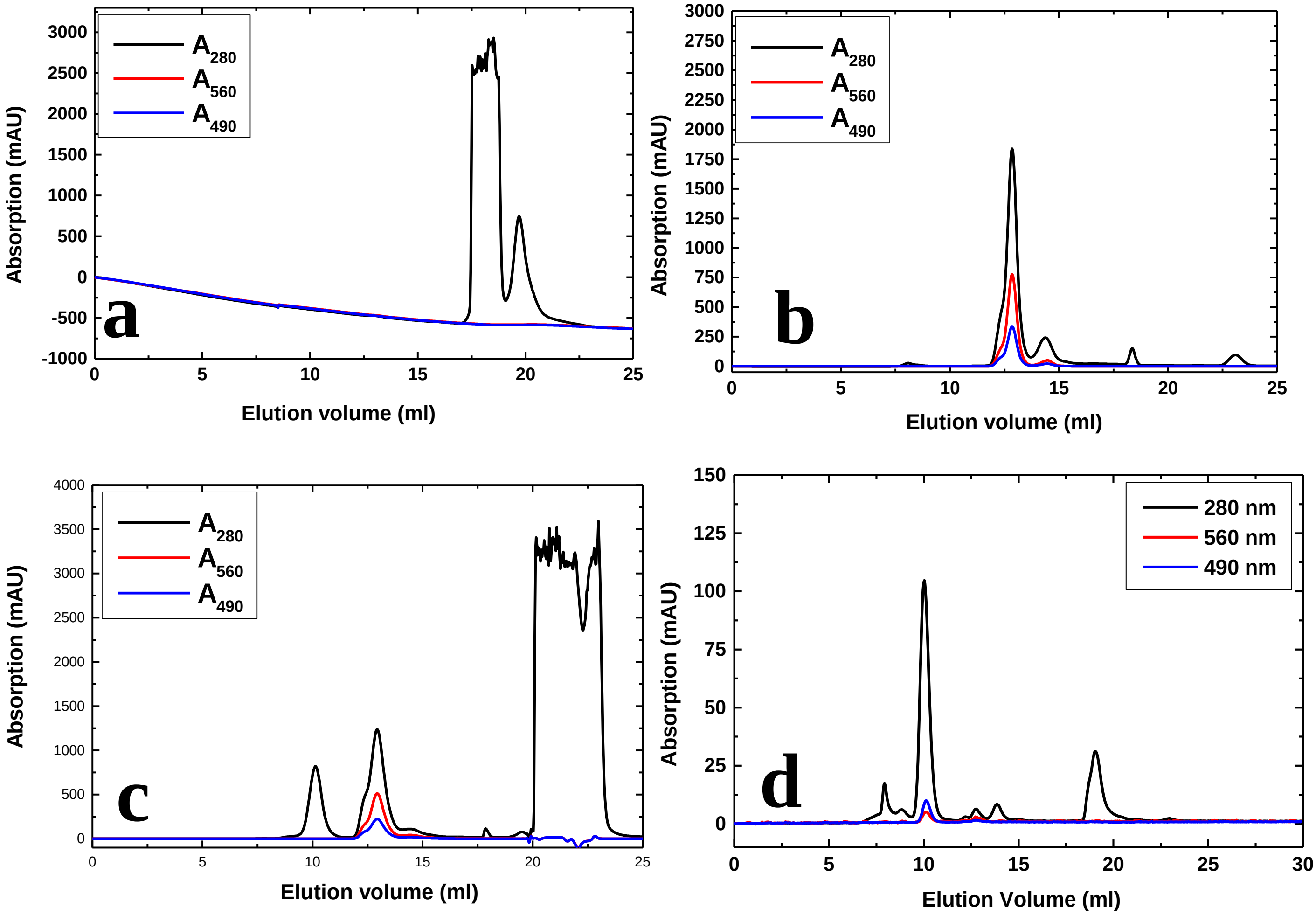

Figure S2

Panel a : Elution of free ferrozine (~17 ml and ~20 ml) on a Superdex Peptide (GE) column in the absence of any iron or protein. **Panel b :** Fe^{2+}-bound ferrozine (two eluting species at 12.5 ml and 14.0 ml) separated from free ferrozine (~17 ml) on the same column. **Panel c:** Fe^{2+}-bound ferrozine (~12.5 ml and ~14.0 ml) separated from free ferrozine (~17 ml and 20 ml) and PfRd protein (~10 ml) on the same column. **Panel d:** Elution of PfRd protein (~10 ml) on a Superdex Peptide (GE) column in the absence of any ferrozine.

DISCUSSION OF FIGURE S3

Confirming that Apo-2 PfRd does not support the formation of any disulfide bonds amongst any of the four cysteines involved in holding the iron atom, once the iron atom is no longer present (and has been removed).

Any formation of disulfide bonds amongst PfRd's cysteine residues could call into question the detailed analytical reasoning and scenario that appears to be emerging from our examinations of the structural-biochemical behavior of Apo-1 PfRd, Apo-2 PfRd and holo-PfRd. Therefore, we decided to examine, in particular, Apo-2 PfRd, the second of the two forms of PfRd which was generated and examined for the very first time in this paper (since Apo-1 PfRd has already been characterized by other authors, albeit using naturally-sourced protein). Our basic approach was to first examine the molecular weight of intact Apo-2 PfRd. This is shown in Supplementary Figure S3a.

Next, as a control, we assumed that there could be some disulfides formed upon departure of the iron atom, in Apo-2 PfRd, and so we first treated it with beta-mercaptoethanol (to reduce disulfides) and then react the cysteine residues with an alkylating agent, iodoacetic acid (IAA). IAA is expected to form adducts with the sulphydryl groups of cysteine residues by displacing any cross-disulfide species involving beta-mercaptoethanol. The mass spectrometric data involving this treated protein is shown in Supplementary Figure S3b.

Finally, we alkylated an Apo-2 PfRd population that had not been pre-treated with beta-mercaptoethanol, so that only free cysteine residues would be seen to react with IAA. The mass spectrometric data for this population is shown in Supplementary Figure S3c. Briefly, the idea was that each alkyl group would increase the mass of PfRd by 57 Da, and that a comparison of the beta-mercaptoethanol unreacted, and reacted, populations would reveal whether any cysteines were non-reactive, either on account of being disulfide-bonded, or being buried within the structure and (therefore) inaccessible to the alkylating agent. To facilitate exposure of all parts of the protein to the alkylating agent, IAA, the alkylation was done at 90 degrees Centrigrade in the presence on 6 M Gdn.HCl, such that the combination of denaturant and temperature would achieve some unfolding of the protein where individually either denaturant or temperature were incapable of doing so.

The result was that with the Apo-2 PfRd population that was never exposed to beta-mercaptoethanol [Supplementary Figure S3c], we were able to see that the population is dominated by species in which all four cysteine residues were accessible to IAA (and thus not engaged in disulfide bonds). In addition, minority populations with three, two and one cysteine alkylated were also seen as would be expected in any such reaction with a protein with hyperthermostable structure. In the mercaptoethanol-treated population too [Supplementary Figure S3b], the dominant population is that of molecules with all four cysteines accessible. The supplementary figures referred to on this page are shown on the next page with legends

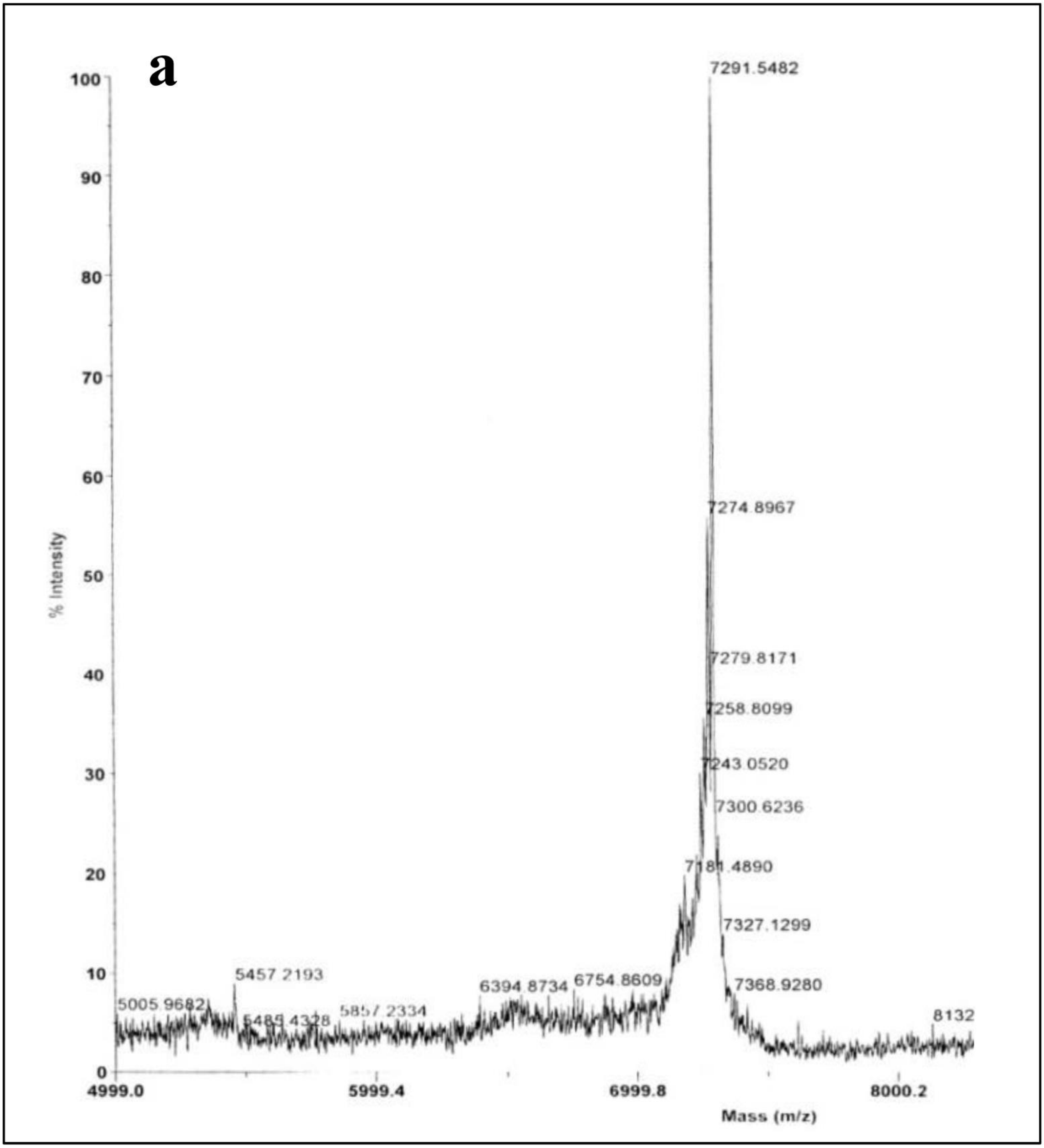

Figure S3. *Panel a :* MALDI-TOF MS spectrum of Apo-2 PfRd (including the N-terminal 6xHis tag), showing that the protein has a mass of 7292 Da. The theoretically expected mass is ~7294 Da.

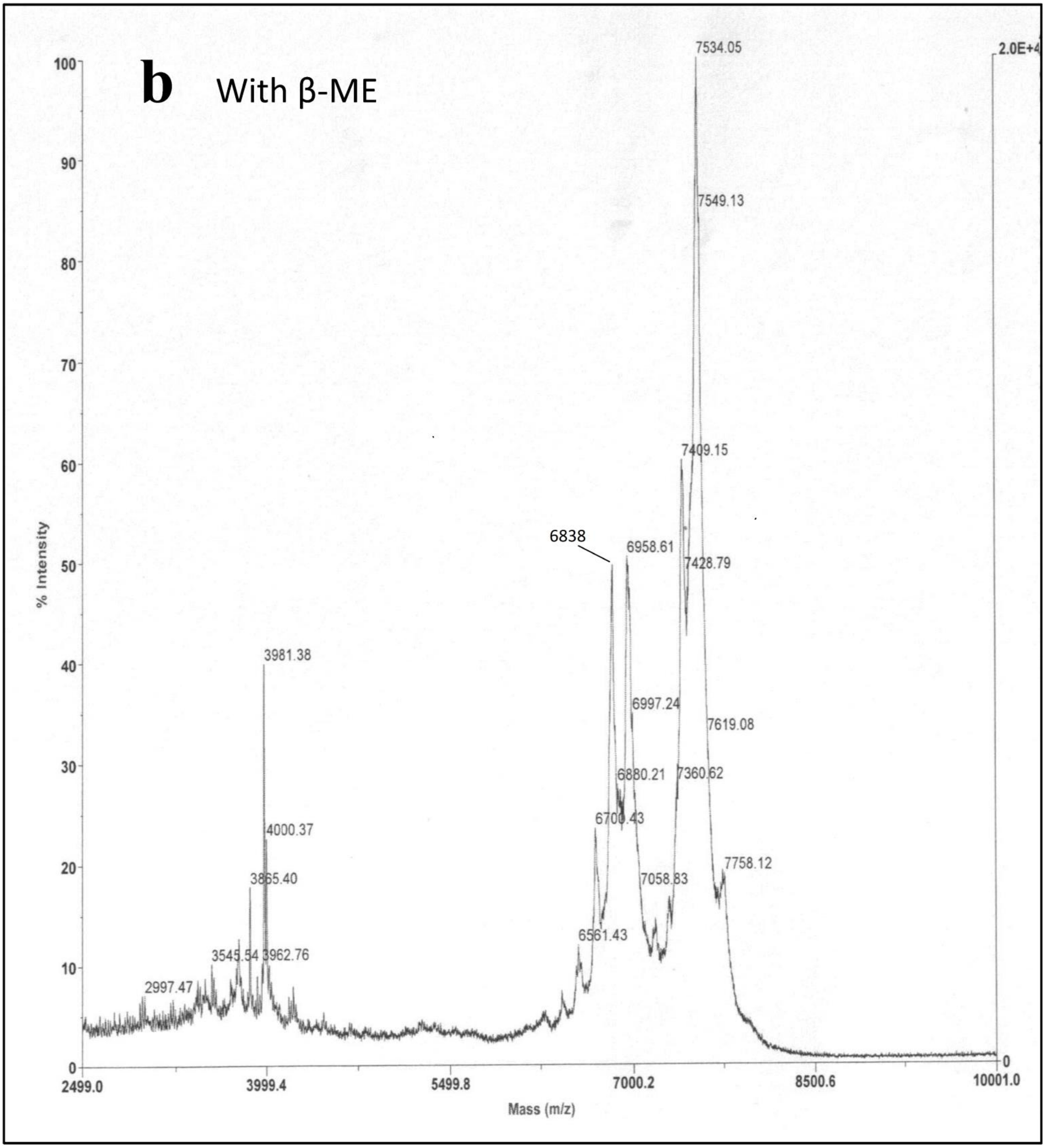

Figure S3. *Panel b* : MALDI-TOF MS spectrum of N-terminally 6xHis tagged Apo-2 PfRd alkylated by iodoacetic acid (IAA) after treatment with beta mercaptoethanol. The masses of 7534, and 7409 Da represent species carrying four, and two, IAA aductions, respectively, indicating that PfRd's four cysteine residues are free and available to be alkylated in the presence of beta-mercaptoethanol.

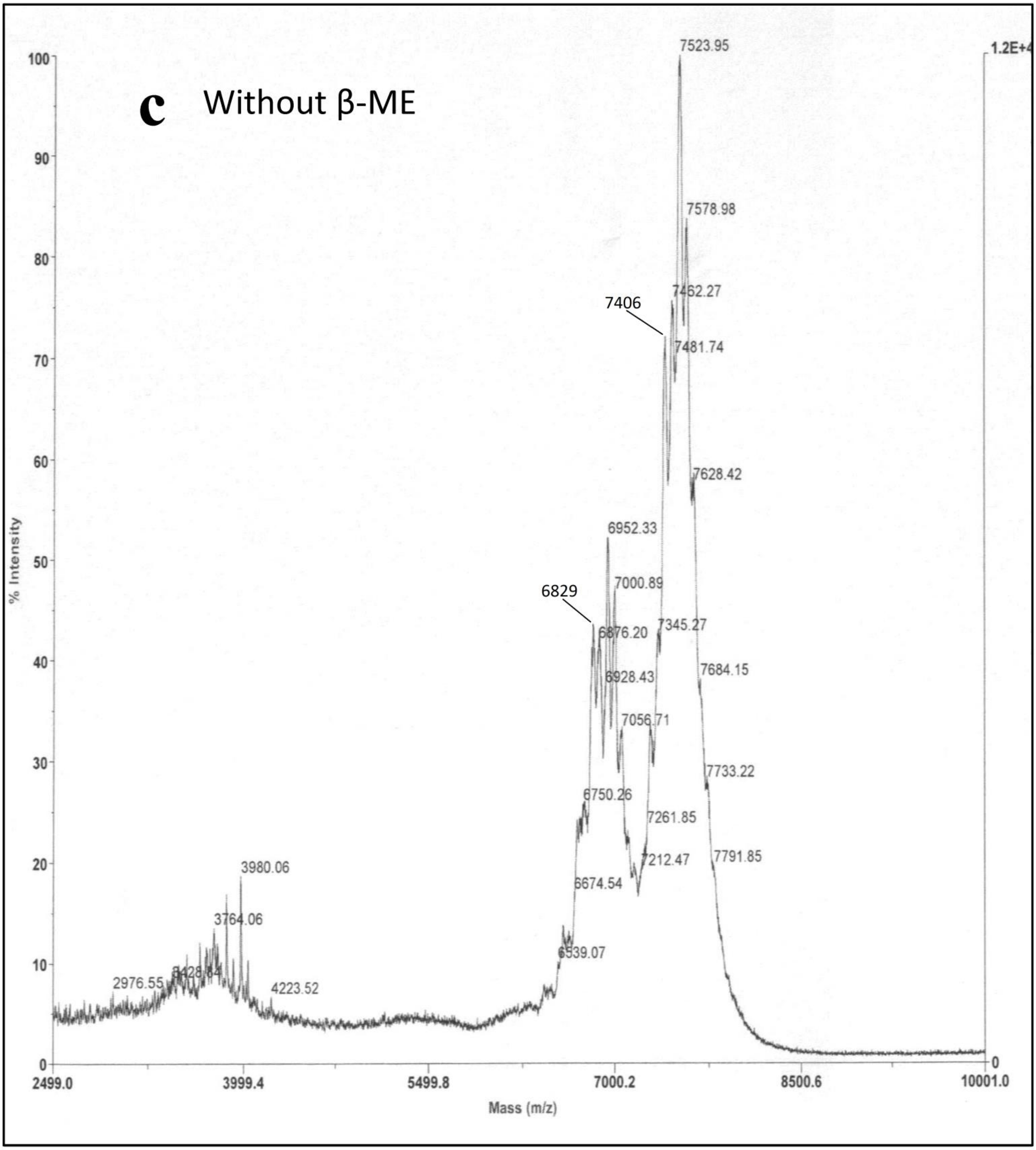

Figure S3. *Panel c* : MALDI-TOF MS spectrum of N-terminally 6xHis tagged Apo-2 PfRd alkylated by iodoacetic acid (IAA) without any treatment with beta mercaptoethanol. The masses of ~7523.95, ~7462.27, ~7406 and ~7345 Da represent species carrying four, three, two, and one IAA aductions, respectively, with the mass peak with the highest intensity representing the population with all four of PfRs's cysteine residues modified. The molecule's cysteine residues are thus free and available to be alkylated (and not disulfide bonded).

DISCUSSION OF FIGURE S4

Structural examination of the stacking, orthogonal and other ring-ring interactions amongst the six aromatic residues of holo-PfRd

We conducted a detailed and thorough examination of the aromatic interactions in the aromatic cluster in the core of holo-PfRd.

Fig. S4 displays all of these interactions in a pair-wise fashion, and also in groups of two or three residues.

From these, figures, it can be seen that residues F29 and W3 interact by ring stacking interactions (Panel b in Fig. S4).

F29 and W36 contact each other through orthogonal ring interactions (Panel c in Fig. S4).

F48 interacts with both F29 and W36 interact (Panel d in Fig. S4).

W36 and Y12 interact with each other (Panel e in Fig. S4).

Y10 and Y12 contact each other (Panel f in Fig. S4).

F48 interacts with both Y10 and W36 (Panel g in Fig. S4).

Therefore, F29 is directly in contact with W3, W36 and F48.

W36 is directly in contact with F29, Y10, Y12 and F48.

No other aromatic residue forms such a huge number of interactions as F29 and W36.

It is clear that F29 and W36 act as key players for this aromatic cluster, almost like the central glue which keeps the cluster together.

The Supporting figures referred to on this page are shown on the next page with legends

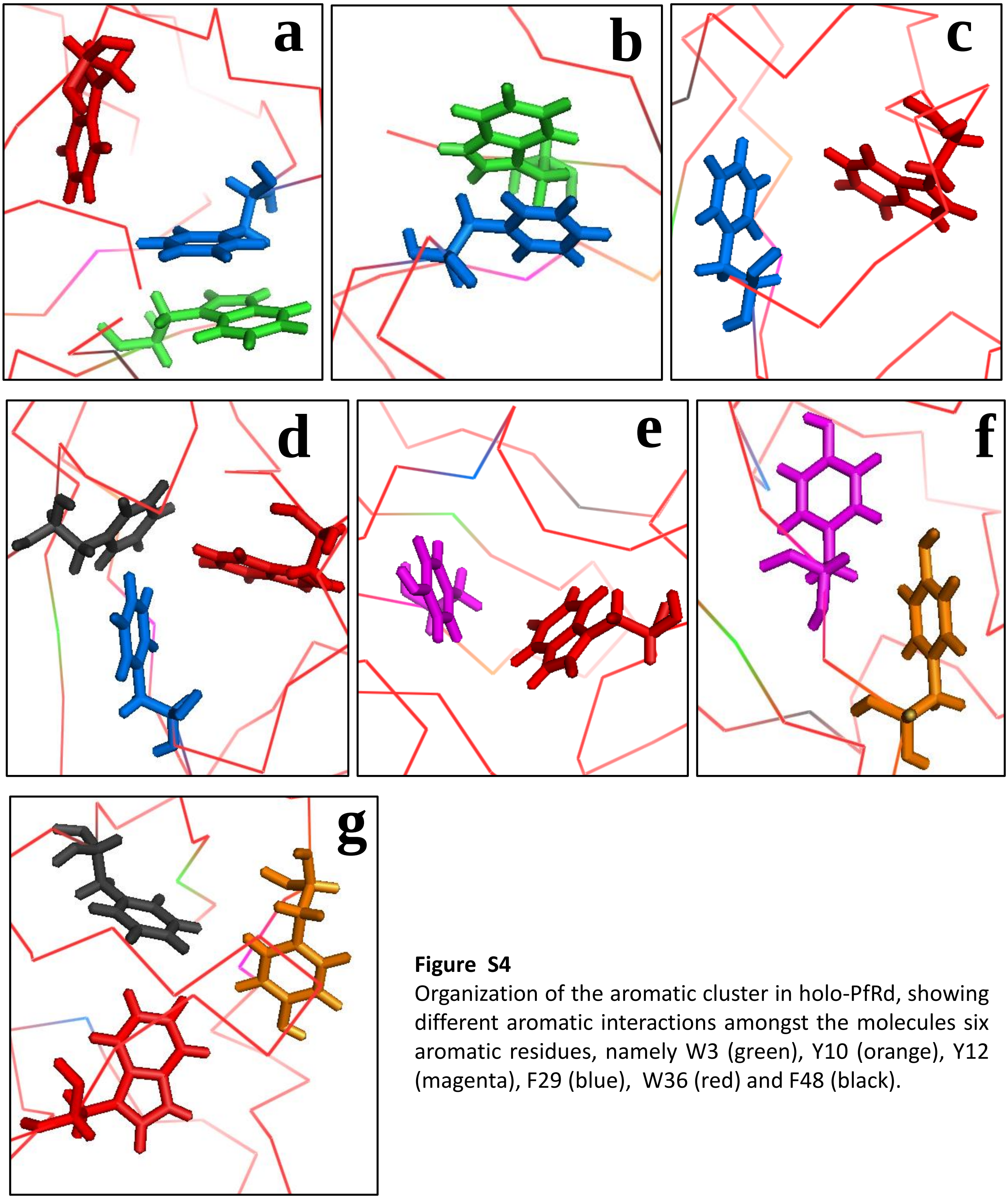

Figure S4
Organization of the aromatic cluster in holo-PfRd, showing different aromatic interactions amongst the molecules six aromatic residues, namely W3 (green), Y10 (orange), Y12 (magenta), F29 (blue), W36 (red) and F48 (black).

DISCUSSION OF FIGURE S5

Examination of changes in the structural characteristics of holo-PfRd and different aliphatic and aromatic residue substitution mutants.

The near-UV and far-UV CD spectra of holo-PfRd and all the variants produced by alanine (or serine) substitute of selected aliphatic and aromatic residues were examined, presented and discussed in Figure 10 in the main paper.

Results from the examination of the corresponding protein species in respect of changes in structural characteristics are presented in Fig. S5, both in respect of proteins heated in the presence and in the absence of denaturant.

Panels a, c and e, respectively, in Figure S5, present data for changes in the far-UV CD signal at 222 nm as a function of rising temperature (panel a), as a function of rising temperature in the presence of 6 M Gdm.HCl (panel c) and as a function of time at a particular temperature of 95 ° C in the presence of the same denaturant (panel e), for all the aliphatic residue substitution mutants.

Panels b, d and f, respectively, present data for changes in the far-UV CD signal at 222 nm as a function of rising temperature (panel b), as a function of rising temperature in the presence of 6 M Gdm.HCl (panel d) and as a function of time at a particular temperature of 95 ° C in the presence of the same denaturant (panel f), for all the aromatic residue substitution mutants.

Collectively, the data reveal the following points :

- Neither holo-PfRd nor any of the aliphatic substitution mutants undergoes any significant unfolding upon heating in the absence of denaturant (panel a). Only mutant I23A loses its additional negative MRE upon heating, such that at 95 °C all variants and holo-PfRd have comparable MRE values. The aromatic substitution mutants have different MRE values to begin with (as discussed in the main manuscript); however, these two do not undergo any changes with heating (panel b).
- Holo-PfRd and all of the aliphatic substitution mutants undergo some thermo-chemical unfolding in the presence of 6 M Gdm.HCl (panel c). With the aromatic substitution mutants, the data is different for different mutants. W3A and Y10A show thermo-chemical unfolding like holo-PfRd. However, W36A and F29A, which have much lower ellipticity to start with, start developing higher negative values of MRE upon heating, while with Y12A and F48A, there is an initial drop in the negative MRE signal, followed by an increase in the negative MRE value (panel d).
- In terms of changes in MRE with time at 95 °C, all the aliphatic substitution mutants display behaviour similar to holo-PfRd (panel e), continuing to display relative slow thermo-chemical denaturation over a time scale of 2500 seconds, whereas with the aromatic substitution mutants (panel f), all mutants differ from holo-PfRd in their behavior. Briefly, with these latter mutants, all changes in MRE that occur (and which are small in extent) are completed within the first 125 seconds of exposure to 95 °C in the presence of 6 M Gdm.HCl.

The Supporting figures referred to on this page are shown on the next page with legends

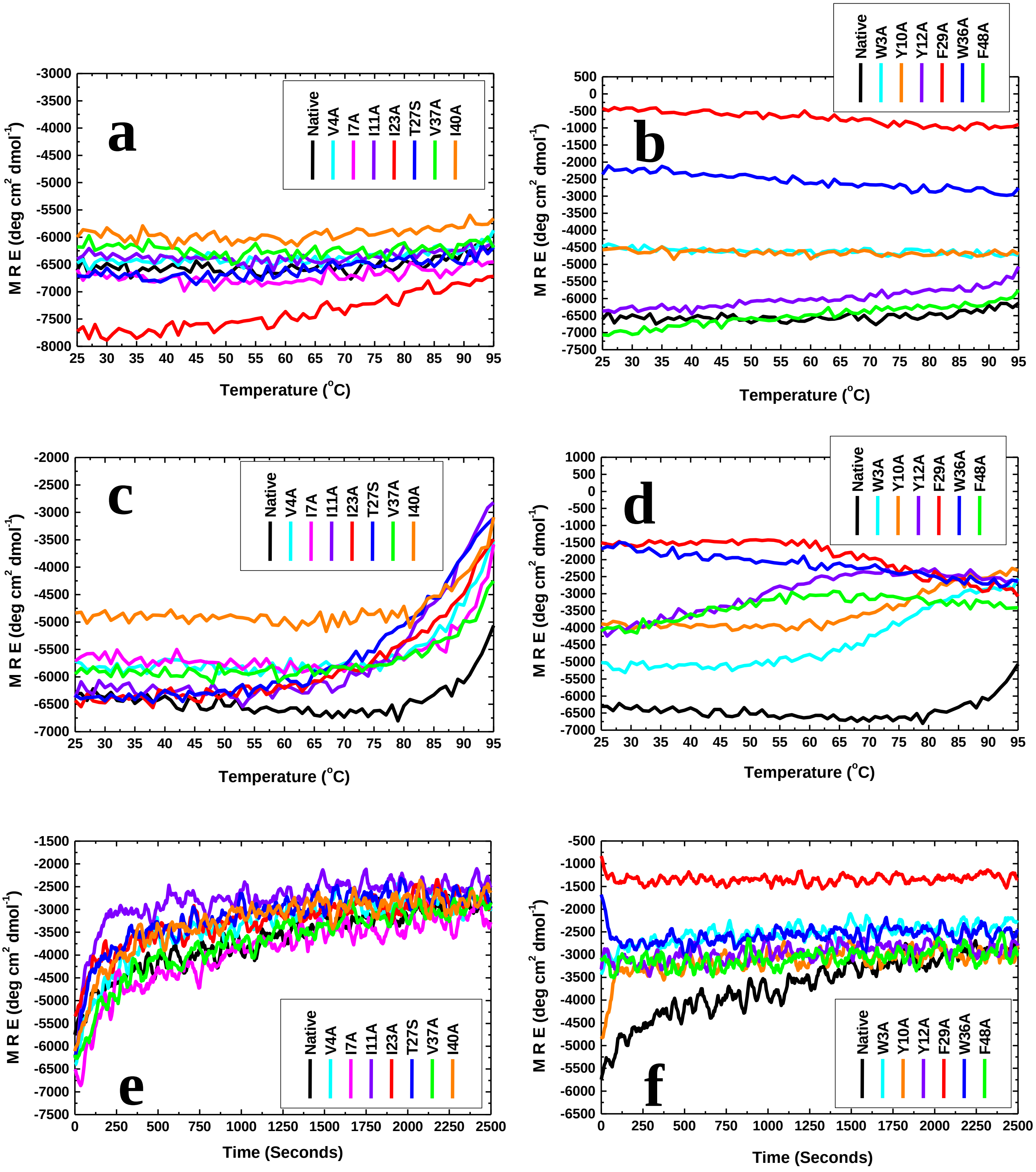

Figure S5

Changes in the CD MRE signal at 222 nm of aliphatic **(Panel a)** and aromatic **(Panel b)** substitution mutants as a function of increasing temperature, in the absence of denaturant. Changes in the CD MRE signal at 222 nm of aliphatic **(Panel c)** and aromatic **(Panel d)** substitution mutants as a function of increasing temperature, in the presence of 6 M Gdm. HCl. Time course of changes in the CD MRE signal at 222 nm of aliphatic **(Panel e)** and aromatic **(Panel f)** substitution mutants at 95 degrees Centigrade, in the presence of 6 M Gdm.HCl.
